# Temperature-Dependent {111}-Texture Transfer to Hf_0.5_Zr_0.5_O_2_ Films from {111}-Textured TiN Electrode and Its Impact on Ferroelectricity

**DOI:** 10.1021/acsami.4c17978

**Published:** 2025-03-28

**Authors:** Dong Hee Han, Seung Yeon Kim, Hyun Woo Jeong, Younghwan Lee, Young Yong Kim, Woojin Jeon, Min Hyuk Park

**Affiliations:** † Department of Materials Science and Engineering & Inter-University Semiconductor Research Center, College of Engineering, Seoul National University, Seoul 08826, Republic of Korea; ‡ Department of Advanced Materials Engineering for Information and Electronics, and Integrated Education Program for Frontier Science & Technology (BK21 Four), Kyung Hee University, Yongin, Gyeonggi 17104, Korea; § School of Materials Science and Engineering, Chonnam National University, Gwangju 61186, Republic of Korea; ∥ Beamline Division, Pohang Accelerator Laboratory, POSTECH, Pohang 37673, Republic of Korea; ⊥ Research Institute of Advanced Materials, Seoul National University, Seoul 08826, Republic of Korea; # Institute of Engineering Research, Seoul National University, Seoul 08826, Republic of Korea

**Keywords:** crystallographic texture, deposition temperature, (Hf,Zr)O_2_, ferroelectric, grazing-incidence wide-angle X-ray scattering

## Abstract

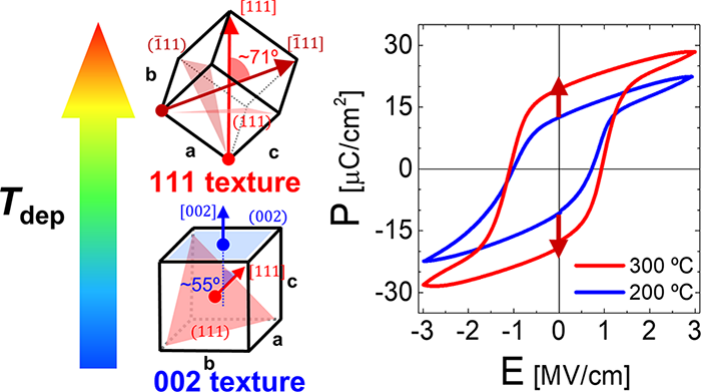

The crystallographic texture of Hf_0.5_Zr_0.5_O_2_ (HZO) thin films plays a crucial role in determining their ferroelectric properties, requiring a deeper understanding of the texture transfer from the substrate. This study investigated the influence of the deposition temperature on the crystallographic texture, residual stress, and ferroelectric properties of HZO thin films. Grazing-incidence wide-angle X-ray scattering analyses confirmed a pronounced increase in the {111} texture of the HZO films when the deposition temperature increased from 200 to 300 °C. The observed {111} texture was attributed to the influence of the thermodynamic stability on in situ nucleation and growth during atomic layer deposition at elevated temperatures, which led to preferential crystallization along the {111} direction. The improved {111}-texture of the HZO film was shown to correlate directly with a ∼25.0% increase in the remanent polarization (*P*_r_) in positive-up-negative-down measurements and a ∼17.2% decrease in the *P*_r_ change during the wake-up effect, reinforcing the superior performance of the films produced at higher temperatures.

## Introduction

(Hf,Zr)O_2_-based ferroelectric thin films have garnered increasing attention from both academia and industry as promising candidates for next-generation nonvolatile memory devices because of their excellent scalability, high compatibility with complementary metal-oxide-semiconductor (CMOS) processes, and superior ferroelectric properties.^1−6^ The formation of the orthorhombic phase (o-phase, space group: *Pca*2_1_) of (Hf,Zr)O_2_ is considered to be the crystallographic origin of the unexpected ferroelectricity, although there is no pressure and temperature condition for the thermodynamic stabilization of bulk (Hf,Zr)O_2_.^7^ The mechanism behind the formation of the metastable ferroelectric o-phase has been debated, but it is generally accepted that various factors affect the polymorphism of ferroelectric (Hf,Zr)O_2_-based thin films, including the doping, film thickness, grain size, deposition conditions, and annealing conditions.^2,8−13^

Because ferroelectricity originates from a broken centrosymmetry, characteristic spontaneous polarization is induced along a specific crystallographic direction, called the polar axis, in noncentrosymmetric crystals. Therefore, the texture of a ferroelectric thin film is a deterministic factor in its ferroelectricity, the characteristic parameters of which are the remanent polarization (*P*_r_) and coercive field (*E*_c_). Ideal ferroelectricity with a maximum *P*_r_ and minimum *E*_c_ can be achieved with single crystals oriented along the polar axis. The deposition of single-crystalline epitaxial films generally requires deposition methods such as pulsed laser deposition (PLD) and molecular beam epitaxy (MBE), as well as a specially chosen substrate.^14−16^ However, for practical semiconductor applications based on CMOS technology, deposition techniques such as PLD and MBE, as well as substrates other than Si, are not considered applicable. Thus, polycrystalline (Hf,Zr)O_2_ films are generally studied for practical semiconductor applications based on deposition methods such as atomic layer deposition (ALD).^7,11,17^

Despite the difficulties in texture control, ALD is currently considered the most promising deposition method for ferroelectric (Hf,Zr)O_2_-based thin films based on the accuracy of the thickness control even for 3D nanostructures, as well as its CMOS compatibility and relatively low deposition temperature.^18−21^ Nonetheless, there is still an urgent need to achieve texture control of a high fraction of the polar axis with ALD to achieve high performance (high *P*_r_, high switching speed, and low *E*_c_) and high reliability (high endurance and retention, as well as low device-to-device and cycle-to-cycle variability). In particular, to develop high-density memory devices based on ferroelectricity, a technology to transfer the texture from the substrate or bottom electrode is urgently needed.

Despite the strong correlation between film texture and ferroelectricity, previous studies have intensively focused on the effects of the (Hf,Zr)O_2_-based film texture. Accurately investigating the texture of films thinner than 10 nm is highly challenging, even with state-of-the-art techniques. Schenk et al. examined the texture and film stress in atomic layer-deposited Si- and La-doped HfO_2_ and showed that the preferred orientation in these films is weak, and they can be considered almost randomly oriented.^22,23^ Park et al. observed a {111}-texture transfer to ferroelectric Hf_0.5_Zr_0.5_O_2_ (HZO) films from the bottom {111}-textured Pt electrode, but because of the unexpected stress effect, the ferroelectricity of these HZO thin films was negligible.^24^ Lee et al. investigated the phenomenon of texture inheritance in a TiN/HZO structure, suggesting that the crystallization of (Hf,Zr)O_2_-based thin films on the {111}- or {200}-TiN surface occurs preferentially along the {111} or (020)/(002) plane, respectively.^21^ Even considering these previous studies, the mechanism behind a texture transfer from the substrate or bottom electrode and an efficient texture-transfer technology are yet to be elucidated.

Therefore, this study investigated a temperature-dependent texture transfer to an HZO thin film from a {111}-textured TiN electrode and its impact on the ferroelectricity. A discrete feeding method (DFM) was employed to suppress the oxidation of the TiN electrode surface by the ALD reactant during the deposition process. Crystal-structure and microstructure analyses of the HZO/TiN stack were used to investigate the causes of the texture formation and differences in texture, and we analyzed how these differences affected the electrical properties of TiN/HZO/TiN capacitors.

## Experimental Section

**Figure 1:**
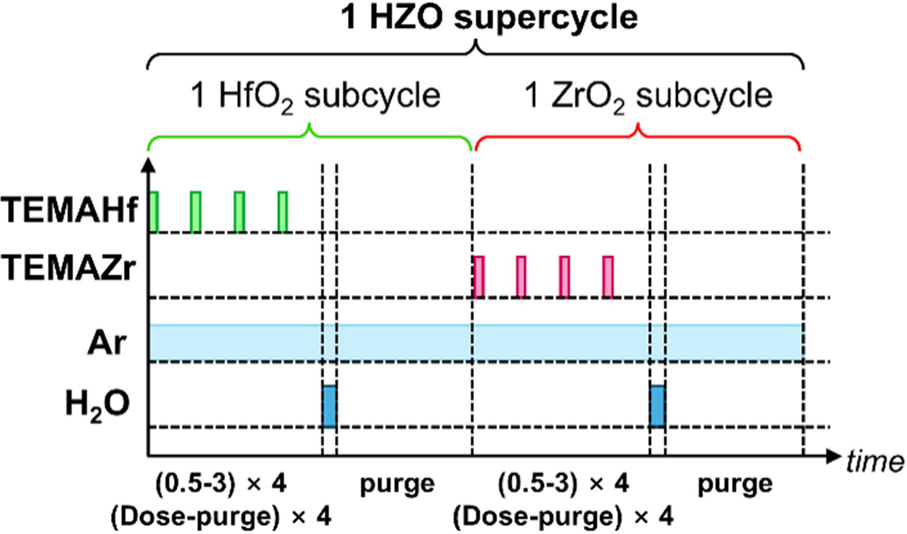
Schematic of DFM-ALD in this study. The deposition sequences are (0.5–3) × 4 – 0.5 – 15 s for (Hf- or Zr-precursor dose – purge) × 4 – H_2_O dose – purge. Alternative HfO_2_ and ZrO_2_ subcycles were used.

A 65 nm-thick TiN bottom electrode was deposited on a Si substrate with native oxide via direct-current (DC) reactive sputtering. The Ar:N_2_ gas flow ratio was adjusted to induce orientation in the TiN, whereas the working pressure and temperature were fixed at 1.5 mTorr and 408 K, respectively. Subsequently, a 10 nm-thick HZO thin film was deposited using a traveling-wave-type ALD system (iSAC research, iOV-mX1). Tetrakis(ethyl-methylamino)-Hf and tetrakis(ethyl-methylamino)-Zr (TEMA-Hf and TEMA-Zr, respectively) were used as the Hf and Zr precursors, respectively, with H_2_O as the oxidizing agent. A DFM was used,^25^ where the precursor dose/purge time was split into four segments. The ALD sequence times for HfO_2_ and ZrO_2_ were (0.5–3) × 4 – 0.5 – 15 s for (Hf- or Zr-precursor dose–purge) × 4 repeats – H_2_O dose – purge. This ALD sequence schematic is present in Figure 1. A 1:1 HfO_2_:ZrO_2_ subcycle ratio was employed to achieve a uniform cation profile in the HZO film. The growth per cycle for both HfO_2_ and ZrO_2_ was approximately 0.12 nm/cycle across the entire deposition temperature range. During the ALD, the chamber pressure was maintained at 800 mTorr.

A 55 nm-thick TiN top electrode was deposited using radio frequency magnetron sputtering with a TiN sputtering target. To measure the electrical properties, a circular top electrode dot with a diameter of 200 μm was formed using a hole-patterned shadow mask. For the crystallization of the HZO film, rapid thermal annealing (RTA) was performed at 700 °C for 10 s in an N_2_ atmosphere. The temperature ramp rate for the RTA process was 14 °C/s. For the structural analysis, a TiN capping layer with the same thickness as the top electrode was deposited and annealed using identical RTA processes. The TiN capping layer was then removed using an SC-1 solution (1:1:5/H_2_O_2_:NH_4_OH:H_2_O) at 65 °C for 2 min.

An X-ray diffraction (XRD) instrument (Empyrean, PANalytical) was employed for Bragg–Brentano geometry XRD and grazing-incidence XRD (GIXRD) (incidence angle of 0.5°) analyses, using Cu Kα radiation with a wavelength of 0.15406 nm as the X-ray source and a point detector. Grazing-incidence wide-angle X-ray scattering (GIWAXS) measurements were conducted at the 3C beamline in PLS-II at the Pohang Accelerator Laboratory, with an incidence angle of 0.5° and incidence beam energy of 11.0 keV (wavelength ∼ 113 pm). A two-dimensional detector (Eiger X4M) was located 214.5 mm downstream of the sample position. Cross-sectional high-resolution transmission electron microscopy (HR-TEM, Thermo Fisher Themis Z system) was conducted at the Research Institute of Advanced Materials (RIAM) to analyze the microstructures of the TiN/HZO/TiN film stacks. The acceleration voltage was set at 300 kV. Electrical characterization of the metal-ferroelectric-metal capacitors was conducted using a semiconductor parameter analyzer (4200A-SCS, Keithley) with a pulse measurement unit (4225-PMU, Keithley) to measure the polarization–electric field (*P*–*E*) curves, along with endurance tests. An LCR meter (4110, Wayne Kerr Electronics) was employed to measure the capacitance–voltage (*C*–*V*) curves.

## Results and Discussion

To induce the {111} texture in the HZO films, a {111}-textured TiN electrode was deposited using DC reactive sputtering. To control the preferred orientation of the TiN, the Ar and N_2_ gas flow ratio was adjusted during DC sputtering process. To deposit {111}-oriented TiN, an Ar:N_2_ flow ratio of 20:5 (standard cubic centimeters per minute) was used, whereas for randomly oriented TiN, an Ar:N_2_ flow ratio of 10:10 was employed after testing various sputtering conditions. The substrate-normal texture-dependence of the sputtered TiN films on the Ar:N_2_ flow rate was analogous to that seen in the process method reported by Greene et al.^26^ The XRD pattern of the {111}-textured TiN bottom electrode is compared to that of the randomly oriented TiN electrode in Figure S1 of the online Supporting Information (SI). In this study, the XRD diffraction peaks from the (*hkl*) planes of the x-phase were labeled as *hkl*_*x*_, where *x* could represent m (monoclinic, space group: *P*2_1_/*c*), o (orthorhombic, space group: *Pca*2_1_), t (tetragonal, space group: *P*4_2_/*nmc*), or TiN.

**Figure 2:**
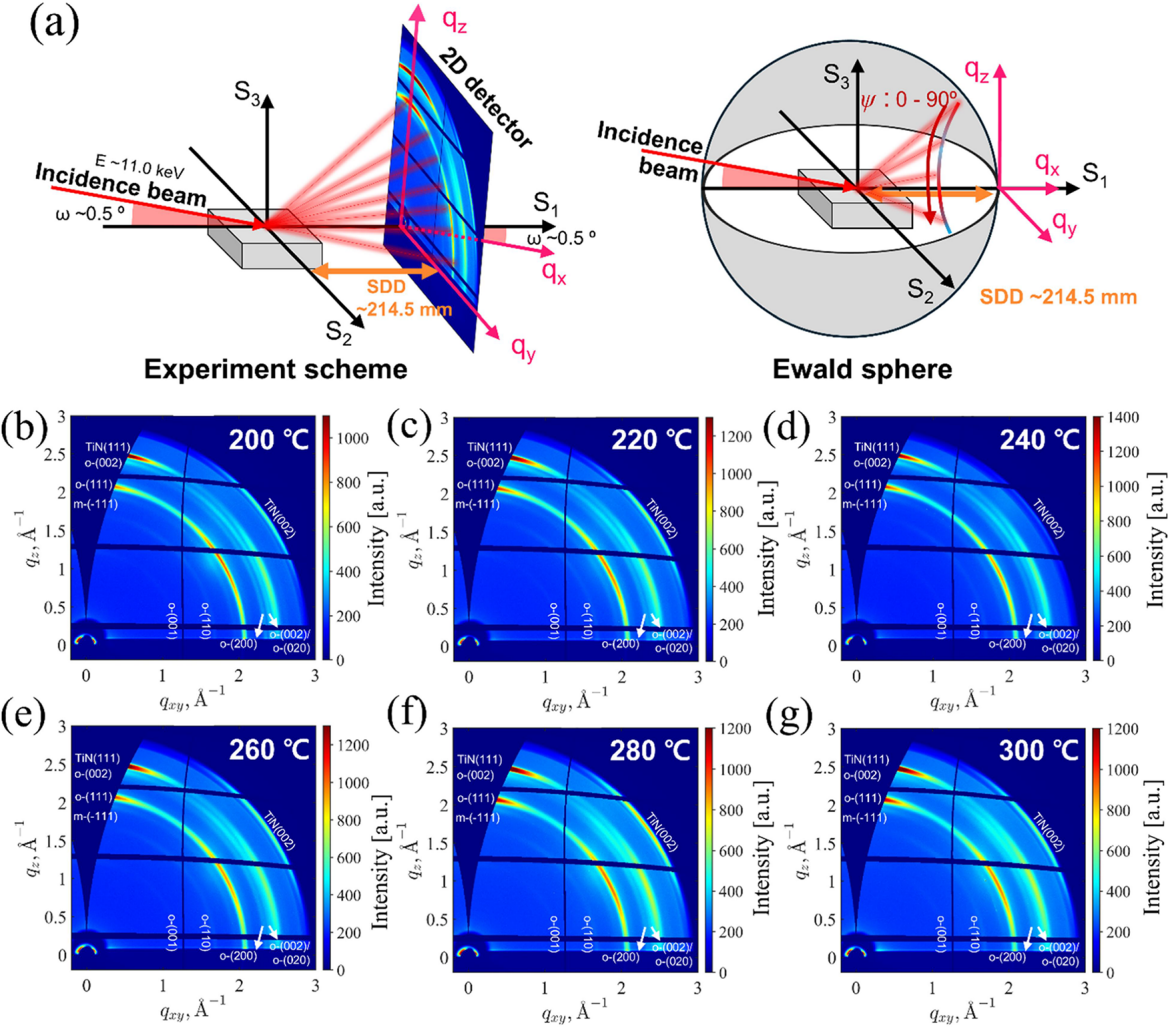
(a) Illustration of the GIWAXS measurement configuration and Ewald sphere. 2D GIWAXS patterns of HZO/TiN stacks when using HZO deposition temperatures of (b) 200, (c) 220, (d) 240, (e) 260, (f) 280, and (g) 300 °C, respectively. (SDD: source–detector distance).

Figure 2a illustrates the GIWAXS configuration and Ewald sphere used in our measurements, while Figure 2b–g show 2D GIWAXS images of HZO films deposited on {111}-textured TiN electrodes at substrate temperatures of 200–300 °C. The annealing temperature of the TiN/HZO/TiN stacks was set to 700 °C to ensure complete crystallization of the HZO films. The GIXRD and XRD patterns obtained using θ–2θ coupled geometry with lab-scale XRD are presented in Figures S2 and S3 in the Supporting Information. After annealing, the TiN capping layer was removed using an SC-1 solution for structural analysis. As shown in Figure 1a, the sample was tilted 0.5° with respect to the incident beam (which had a wavelength of ∼113 pm). The GIWAXS images were calibrated using p-GIXS software,^27^ which transformed the diffraction intensity originally represented in a 2D plane into a *q*_*xy*_–*q*_*z*_ sphere form. This transformation allowed for the accurate mapping of the diffraction pattern in reciprocal space, providing more detailed information about the crystallographic structure of the sample. This capability enables GIWAXS to provide comprehensive crystallographic information, including preferred orientation and in-plane structural details, which are challenging to capture using conventional XRD or other techniques.

Various Debye–Scherrer rings can be observed in the 2D GIWAXS images in Figure 1, including m-{-111} (2θ ∼ 28.5°), o-{111}/t-{101} (2θ ∼ 30.5°), and o-(002)/o-(020)/t-{110}(2θ ∼ 35.5°) from the HZO film, and {111} (2θ ∼ 36.7°) and {002} (2θ ∼ 42.6°) from the cubic TiN electrode. It should be noted that the maximum 2θ value was limited to 41° because of the photon energy and sample-to-detector distance. The negligible intensity of the m-{-111} Debye–Scherrer ring confirmed that the monoclinic phase (m-phase, space group: *P*2_1_/*c*) formation could be effectively suppressed at all deposition temperatures. The suppression of the m-phase formation could also be confirmed by the Bragg–Brentano and grazing incidence geometry XRD patterns shown in Figures S2 and S3, respectively. The highest intensity along the out-of-plane direction could be observed from the o-{111}/t-{101} Debye–Scherrer rings in the 2D GIWAXS images of all the HZO films deposited at 200–300 °C, which was consistent with the surface-normal {111}-texture of the HZO film and TiN electrode observed in the diffraction patterns of the HZO/TiN stacks measured with the Bragg–Brentano geometry, as shown in Figure S2g. Although the X-ray diffraction by atomic planes normal to the substrate was not directly examined within the GIWAXS measurements because of the Ewald sphere effect and incidence angle,^28^ it is generally accepted that the azimuthal-angle (ψ)-dependence of the intensity can be fitted using a pseudo-Voigt model.^29^

**Figure 3:**
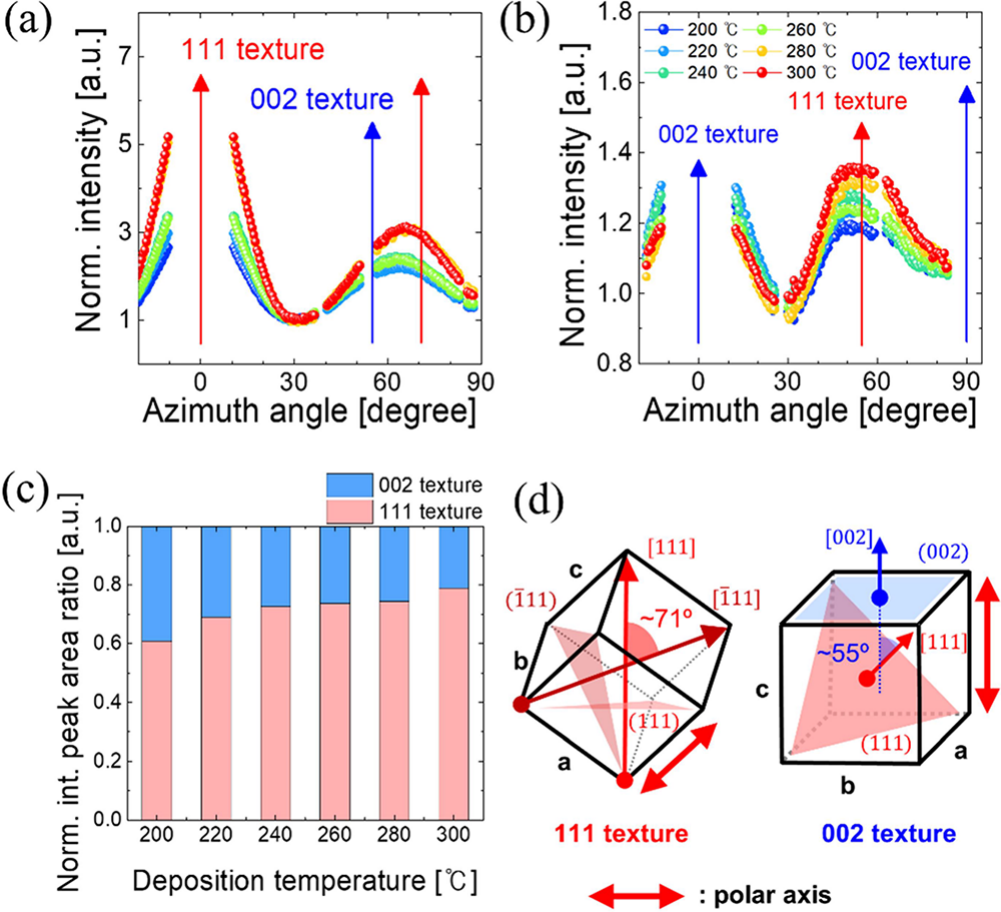
Normalized intensities extracted along ψ from the HZO/TiN GIWAXS images at *q* ∼ 2.11 Å^–1^ and *q* ∼ 2.45 Å^–1^, corresponding to (a) o-{111}/t-{101} and (b) o-(002)/o-(020)/t-{110}diffractions, respectively. (c) Relative areal intensity ratios for o-{111}/t-{101} diffraction originating from the o-(002)/o-(020) and o-{111} textured parts in the HZO films. (d) Schematic of 111 and 002 textures, where the angle between [111] and [−111] was approximately 71° and that between [111] (and [−111]) and [002] was approximately 55°.

Figure 3a,b show the intensity variations according to ψ for the o-{111}/t-{101} and o-(002)/o-(020)/t-{110} Debye–Scherrer rings, respectively. These intensity profiles were extracted from the *q*_*xy*_–*q*_*z*_ plots of the HZO/TiN GIWAXS images at *q* ∼ 2.11 ± 0.1 Å^–1^ and *q* ∼ 2.45 ± 0.1 Å^–1^ using the p-GIXS software and were normalized at 30° for o-{111} and 87° for o-(002)/o-(020). The normalization at 30° for o-{111} was performed because this region is minimally influenced by the contributions of both the {111} and (002)/(020) textures, allowing for a more accurate comparison of the azimuthal intensity variations. Similarly, the normalization at ψ = 87° for o-(002)/o-(020) was selected because this region is the most reliable reference point for these reflections in our GIWAXS measurements. Unlike the o-{111} planes, which are affected by missing wedge effects that hinder accurate intensity quantification in the ψ = 0° direction, the o-(002)/o-(020) diffractions near ψ = 87° correspond to the symmetry of the ψ = 90° direction and provide stable intensity data. However, the diffraction at ψ = 90° cannot be directly observed due to the limitations imposed by the sample tilt and the 2D detector geometry in our GIWAXS setup. If HZO had a random orientation, the intensity would remain roughly constant across ψ in the Debye–Scherrer rings. However, variations in the diffraction intensity with respect to ψ would indicate that the thin film had a preferred orientation. Additionally, the (002)/(020)/(200) peak intensities are inherently weaker due to their structure factor (HfO_2_, orthorhombic, *Pca*2_1_, PDF No.: 04-005-5597), which results in their intensities being only 12–14% of the o-{111} peak intensity. This reduced intensity further complicates peak deconvolution, particularly when overlapping signals or noise are present. In this case, TiN (111) peak of *q* ∼ 2.53 Å^–1^ overlaps with o-(020)/(002), making the separation of these peaks even more challenging. Furthermore, due to the nature of GIWAXS geometry and the interaction with the Ewald sphere, the detection of (002)/(020)/(200) peaks is more sensitive to grain orientations. This makes it even more challenging to accurately separate their intensity variations along the azimuthal angle, leading to additional complexity in deconvolution.

Both the HZO and TiN films measured in this study exhibited a preferred orientation along the ⟨111⟩-direction, with *q* ∼ 2.11 Å^–1^ for the HZO films and *q* ∼ 2.53 Å^–1^ for the TiN films. As shown in Figure S4, one notable observation was that the HZO films grown on randomly oriented TiN displayed a random orientation, with a nearly uniform intensity across ψ at a *q* value corresponding to o-{111}/t-{101}. Lee et al. reported that HZO films could inherit the preferred orientation of the underlying {111}- or {200}-textured TiN, and proposed two possible pathways for texture inheritance in HZO/TiN.^21^ First, nucleation can occur on the {111)-oriented TiN during the ALD process, leading to crystallization along the {111} plane with the lowest surface energy. Second, crystallization into the {111} plane can occur during the annealing step owing to its low surface energy.

As seen in the normalized intensity graph extracted at *q* ∼ 2.11 Å^–1^ (Figure 3a), the intensity near ψ = 55° does not exhibit a systematic decrease with increasing deposition temperature. Instead, the data suggest that the distribution of intensity along the azimuthal angle changes subtly with deposition temperature. In the pseudocubic reference frame, the angle between the {111} and (002)/(020) planes was approximately 55°, and the angles between different members of the {111} family were approximately 71°. Therefore, when the {111} plane grew in the out-of-plane direction, the Debye–Scherrer ring corresponding to the *q* value for {111} exhibited diffractions at azimuth angles of −55° and +55° for the (002)/(020) diffraction, and −71° and +71° for diffractions from the {111} family. Consequently, the peak shift toward higher ψ values in the 60–70° range observed in Figure 3a resulted from the suppression of (002)/(020)-oriented crystallization along the out-of-plane direction, as well as the increasing crystallization along the {111} direction with higher deposition temperatures. To better quantify the observed changes, we analyzed the relative fractions of various substrate-normal textures for the examined deposition temperature based on the deconvolution fitting for the intensity variation with respect to the azimuth angle at *q* ∼ 2.11 Å^–1^ from the GIWAXS image symmetrized around 0° in Figure S5 of Supporting Information. Figure 3c shows the relative fractions of various substrate-normal textures based on the deconvolution fitting. In this plot, the intensity changes along the azimuthal angle are deconvoluted into three peaks in Figure S5: o-(111) diffraction originating from the o-(111) textured part, o-(1̅11) diffraction originating from the o-(111) textured part at 71°, and o-{111} diffraction originating from the o-(002)/o-(020) textured part at 55°. The crystallographic relationships are shown in Figure 3d. The deconvoluted peaks were fitted to a pseudo-Voigt distribution function. The fitting parameters are provided in the Supporting Information (Figure S5 and Table S1). As the deposition temperature increased, the full-width at half-maximum (fwhm) of the out-of-plane o-{111} peak decreased, while the intensity and fwhm values of the o-(002)/o-(020) peaks at −55° and +55° increased. This indicated that the {111} orientation of the crystallized HZO became more dominant as the deposition temperature increased. The normalized integrated peak area listed in Table S2 further confirms that the relative fraction of the o-(1̅11) diffraction originating from the o-(111)-textured part increased with the deposition temperature, whereas the relative fraction of the o-{111} diffraction originating from the o-(002)/o-(020)-textured part decreased.

In contrast, the normalized intensity graph extracted at *q* ∼ 2.45 Å^–1^ (Figure 3b) showed that the o-(002)/o-(020) diffraction in the out-of-plane direction decreased with increasing deposition temperature. This was consistent with the lab-scale GIXRD results shown in Figure S6, where nuclei and/or grains formed at higher deposition temperatures preferentially grew along the [111] direction owing to their lower surface energy, leading to the suppression of surface-normal growth along the o-[002]/o-[020] direction.

**Figure 4:**
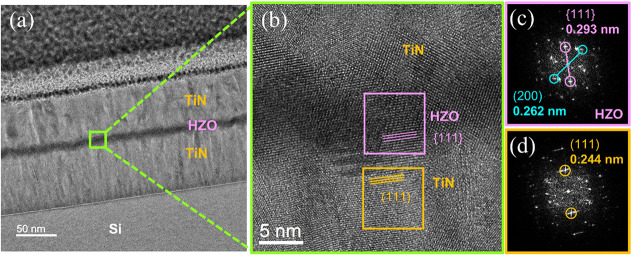
Cross-sectional (a) TEM and (b) HR-TEM images. (c) and (d) show FFT images of the HR-TEM image, where (c) shows the HZO region and (d) shows the TiN region with an HZO deposition temperature of 300 °C.

As shown in Figure 4, cross sections of the TiN/HZO/TiN capacitor were analyzed using high-resolution transmission electron microscopy (HR-TEM) to determine the structural characteristics and interface formation. The deposition temperature of the HZO film analyzed by using TEM was 300 °C. The thicknesses of the {111}-oriented TiN bottom electrode, HZO film, and TiN capping layer were approximately 65, 10, and 55 nm, respectively. The HR-TEM analysis revealed that interfacial oxides such as TiO_2_ and TiO_*x*_N_*y*_ were scarcely observed. This suggested that the introduction of the DFM ALD process effectively suppressed the formation of interfacial oxides, as suggested in previous studies.^30,31^ This suppression of interfacial oxidation is expected to positively influence the electrical performance of a device. Specifically, the surface oxidation of an electrode can increase its resistance and cause voltage drops across the interfacial layer, which reduces the effective voltage applied to the ferroelectric material, thereby slowing the ferroelectric switching behavior.^18,32−34^ The effect of ALD oxidant such as H_2_O and O_3_ on {111}-texture transfer between the TiN and the HZO films was investigated and described in Figure S7.

The fast Fourier transform (FFT) patterns obtained in the designated regions of Figure 4c,d showed *d*-spacing values corresponding to the {111} plane for both HZO (0.293 nm) and TiN (0.211 nm). Notably, the diffraction spots for both HZO and TiN were aligned with an angle difference smaller than 3°, which confirmed the texture inheritance between the TiN and HZO. This preferred {111} orientation of HZO would contribute to the maintenance of consistent electrical properties within a device. While nonoriented films may exhibit local variations in electrical properties, a consistently oriented crystal structure along the ⟨111⟩ direction would ensure superior device performance compared to that of randomly oriented films, which would also be beneficial for achieving low device-to-device variability when integrated into practical semiconductor devices.

To investigate the mechanical stress evolution in HZO films during texture transfer, a modified sin^2^Ψ analysis was conducted based on GIWAXS measurements. The residual stress was evaluated by extracting the o-{111} diffraction peak positions and applying a geometric correction factor. The analysis revealed that as the deposition temperature increased from 200 to 300 °C, the in-plane normal stress decreased, while the residual shear stress increased. This trend suggests that the strengthening of the {111} texture correlates with an increase in shear stress.

Although the exact origin of the observed shear stress has yet to be elucidated, it could be attributed to the martensitic phase transformation from the m-phase to the o-phase during rapid thermal annealing process. A detailed description of the residual stress analysis with modified sin^2^Ψ method based on the GIWAXS analysis, is provided in the online SI, which includes Figures S8 and S9. Also, the obtained residual normal and shear stresses from the modified sin^2^Ψ method are presented in Table S3.

**Figure 5:**
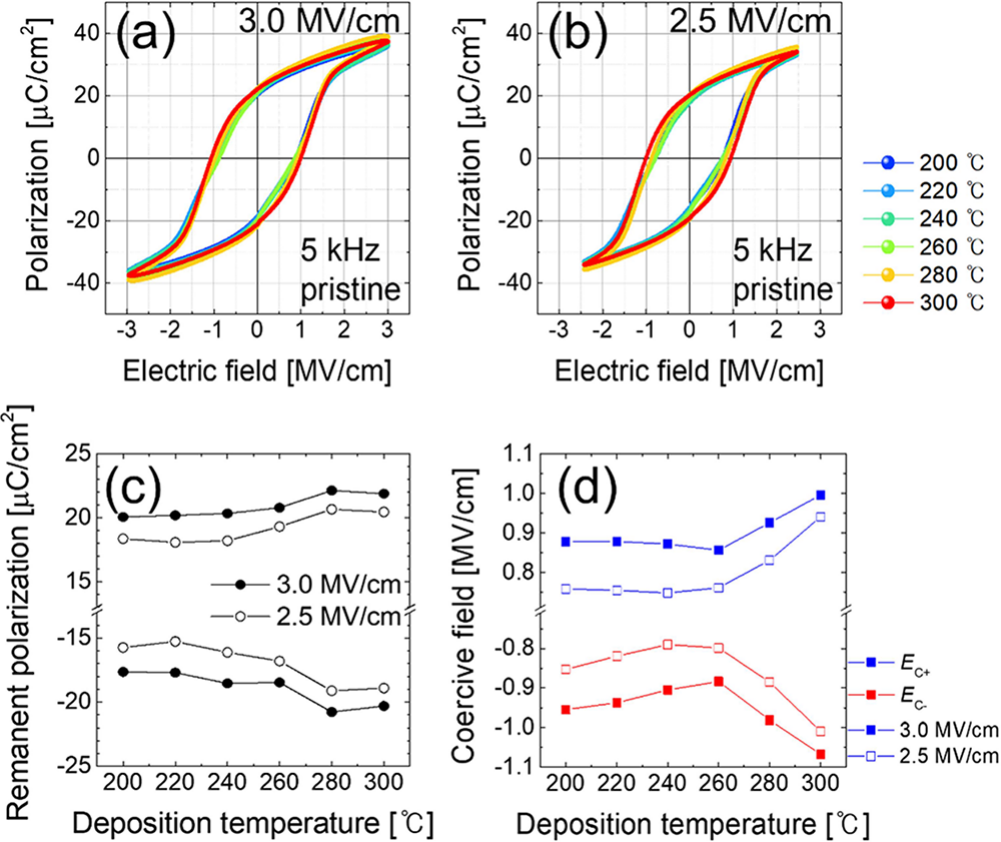
Polarization versus electric field (*P*–*E*) curves of (a) E-fields of 3.0 and (b) 2.5 MV/cm. (c) Remanent polarization (*P*_r_) values of HZO films with different deposition temperatures. (d) Coercive field (*E*_c_) values extracted from measured *P*–*E* curves.

Figure 5a,b show *P*–*E* curves based on the quasi-static hysteresis measurements performed at electric fields (E-fields) of 3.0 and 2.5 MV/cm, respectively, with a bipolar triangular electric field at a frequency of 5 kHz. The *P*–*E* curves for various HZO deposition and annealing temperatures are shown in Figure S10 in online SI. The transient *P*–*E* and current density versus electric field (*J*–*E*) curves with various pulse frequency are shown in Figure S11. Figure 5c shows the *P*_r_ values as a function of the deposition temperature for each E-field, demonstrating a clear increase in *P*_r_ with the deposition temperature. The 2*P*_r_ value increased by 12%, from 37.7 μC/cm^2^ at a deposition temperature of 200 °C to 42.2 μC/cm^2^ at a deposition temperature of 300 °C. The changes in the *P*–*E* hysteresis depending on the deposition temperature were closely related to the differences in the microstructures of the HZO films caused by the thermally activated processes. The dielectric constant-electric field curves of the TiN/HZO/TiN capacitors and deposition temperature-dependent evolution of the dielectric constant are shown in Figure S12 in the online SI. As confirmed by the GIWAXS results in Figure 2, TEM analysis results in Figure 4, and XRD results in Figure S2, as the deposition temperature increased, the relative fraction of the {111}-oriented region increased, whereas that of the o-(002)/o-(020)/t-{110}-oriented region decreased. This change in the direction of crystallographic growth significantly affected the alignment of the polarization axis, contributing to more uniform and higher area-averaged *P*_r_ values. Because the HZO films grown along the ⟨111⟩ direction exhibited uniform polarization across all symmetry planes, they showed a higher overall *P*_r_. Figure 5d shows the *E*_c_ values extracted from the hysteresis curves measured in Figure 5a,b, which showed a trend to increase with the deposition temperature. It is possible that the relative fraction of the m-phase, which exhibited low-*k* characteristics, increased with the deposition temperature. This could have led to a reduction in the effective electric field applied to the o-phase, thereby requiring a higher *E* field to induce the same polarization reversal.

**Figure 6:**
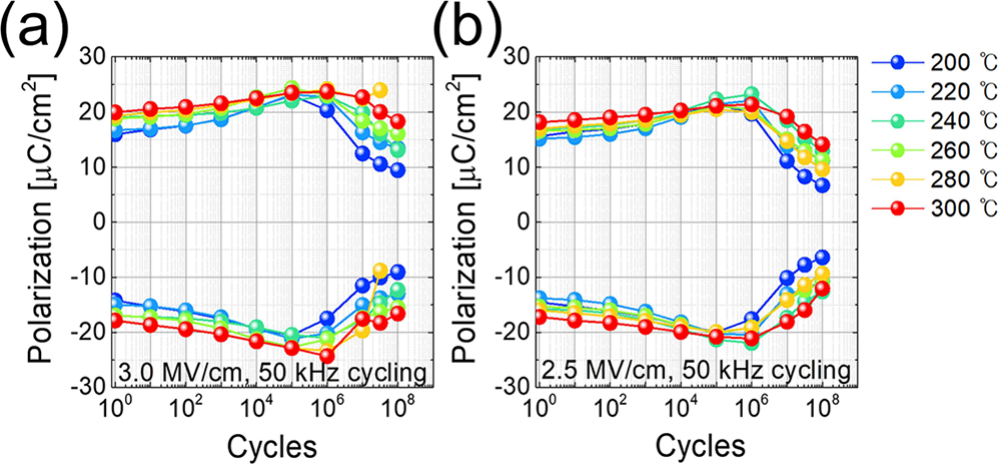
Endurance test results for the HZO films with different deposition temperatures. Rectangular electric field pulse cycling amplitudes of (a) 3.0 and (b) 2.5 MV/cm were used, where the cycling frequency was 50 kHz, and *P*_r_ was measured using a 1 kHz PUND pulse scheme.

Figure 6 shows the endurance test results for TiN/HZO/TiN capacitors obtained using positive-up-negative-down (PUND) pulses. PUND measurements are beneficial for evaluating pure ferroelectric polarization switching in a device, excluding the effects of leakage current or dielectric components. The PUND pulses were measured using triangular pulses at 1 kHz, with bipolar rectangular pulses having amplitudes of 3.0 and 2.5 MV/cm applied for up to 10^8^ fatigue cycles. The endurance test results indicated that as the deposition temperature increased, the 2*P*_r_ value in the pristine state increased by 25.0%, from 30.2 μC/cm^2^ at 200 °C to 37.8 μC/cm^2^ at 300 °C, while the wake-up effect, where 2*P*_r_ increased during cycling, decreased. The changes in the normalized 2*P*_r_ with respect to the maximum 2*P*_r_ value observed during the endurance test are shown in Figure S13 in the online SI.

The larger 2*P*_r_ increase during wake-up cycling in the HZO films deposited at lower temperatures could have been due to the phase transformation from the tetragonal phase (t-phase, space group: *P*4_2_/*nmc*) to the ferroelectric o-phase during field cycling. The other potential origin is the orientation evolution from the (200)/(020)-oriented region to the polar (002)-oriented region, which could have been due to the motion of the low-angle grain boundaries between neighboring (020)/(200)-textured crystallites. Grimley et al.^34,35^ reported the coexistence of (200)-, (020)-, and (002)-oriented domains, even in the grains of Gd-doped HfO_2_ thin films. The rotational transition between the (200)-, (020)-, and (002)-oriented domains is very possible in HZO films deposited at temperatures near 200 °C, although this mechanism requires further study. This would result in domain structure changes that contribute to a more pronounced wake-up effect with an enhanced 2*P*_r_ increase in the HZO films deposited at temperatures near 200 °C. The XRD patterns relevant to the above discussion, with intensities extracted at 5° increments of Ψ from 0° to 90°, are presented in Figure S9 of the online SI. A detailed description is also provided in the online SI.

Another noticeable positive effect of an elevated deposition temperature is the mitigation of fatigue, for which the typical evidence is a decrease in *P*_r_ after the wake-up effect. As shown in Figure 5a, 2*P*_r_ decreased by ∼51.1%, from 37.8 to 18.5 μC/cm^2^, for the TiN/HZO/TiN capacitor with a deposition temperature of 200 °C when the switching cycle number increased from 10^6^ to 10^8^. However, in the case of the TiN/HZO/TiN capacitor with a deposition temperature of 300 °C, 2*P*_r_ decreased by ∼27.4%, from 48.1 to 34.9 μC/cm^2^, within the same switching cycle number range. While the TiN/HZO/TiN capacitor deposited at 300 °C exhibits improved fatigue resistance. From our current understanding, the enhanced texture would decrease the density of defects such as grain boundaries in polycrystalline films. However, it is not certain whether this improvement is solely attributed to the stronger {111} texture. Generally, fatigue is known to originate from the pinning of domain walls by chemical impurities or crystallographic defects, as well as from the formation of the antipolar *Pbca* orthorhombic phase.^36^ Kim et al.^37,38^ reported that a decreased deposition temperature increases the concentration of residual impurities such as C and N, which can induce domain wall pinning.

## Conclusions

This study investigated a temperature-dependent {111}-texture transfer from a {111}-textured TiN bottom electrode to HZO films deposited using the DFM and an H_2_O oxygen source to mitigate interfacial oxidation with a resulting enhancement of the texture transfer. GIWAXS analyses revealed that as the deposition temperature increased, the {111} orientation of the HZO film improved, whereas the (020)/(002) orientation weakened simultaneously. This was attributed to the promotion of {111}-oriented nuclei formation at higher deposition temperatures during the HZO deposition process. The higher deposition temperature also enhanced the ferroelectric properties and reliability of the HZO, particularly in terms of *P*_r_ (which increased by 12.0% when the deposition temperature increased from 200 to 300 °C) and a weakened wake-up effect, with a ∼17.2% reduction in the *P*_r_ increase during electric field cycling up to 10^6^ cycles. The possible reasons for the weakened wake-up effect with an increase in the deposition temperature were the decreased phase transition from the t-phase to o-phase due to the enhanced o-phase fraction and the decrease in the rotational orientation evolution due to the decreased fraction of the (200)- and (020)-oriented domains.

These findings indicated that high-temperature deposition and the adoption of the DFM and H_2_O oxygen source hold promise for optimizing the performance of HZO-based ferroelectric devices with high 2*P*_r_ values and weakened wake-up effects. Additionally, the correlation between the residual stress and phase transformation was confirmed through a stress analysis based on a modified version of the sin^2^Ψ method. It could be confirmed that the in-plane normal stress was released, while the shear stress increased with an increase in the deposition temperature from 200 to 300 °C. This suggested that the martensitic transformation from the initial m-phase nanocrystallites to the final ferroelectric o-phase with an intermediate transition t-phase would affect the evolution of both the normal and shear stresses. This comprehensive approach aimed to provide important insights into the optimization of the deposition temperature to achieve crystallographic texture formation in HZO thin films, along with superior device performances.

## Supporting Information Available

Scheme and patterns of X-ray diffraction within Bragg–Brentano geometry, XRD patterns obtained using theta-2theta coupled geometry for the HZO/TiN stacks annealed at 400–700 °C with HZO deposition temperatures of 200–300 °C, XRD patterns obtained using glazing incidence geometry with omega angle of 0.5° for the HZO/TiN stacks annealed at 400–700 °C with HZO deposition temperatures of 200–300 °C, GIWAXS 2D images of HZO film with randomly oriented TiN and HZO film with a (111)-oriented TiN, where the HZO deposition temperature was 240 °C, normalized intensity plots according to azimuth angle at *q* ∼ 2.11 Å of o-{111} for HZO deposition temperatures of 200–300 °C, fitting parameters of o-{111} azimuthal analysis where fitting was conducted using a pseudo-Voigt distribution function, normalized integrated peak areal ratios of the deconvoluted peaks, GIXRD patterns of the same HZO/TiN stacks before crystallization at these deposition temperatures and normalized intensity ratio of seed and TiN(111) diffractions, GIXRD and theta-2theta coupled geometry XRD patterns of the HZO/TiN stacks where H_2_O and O_3_ were used as reactants, derivation of the equation for stress analysis, stress analysis results using modified sin^2^Ψ method with GIWAXS measurements, magnitudes of the in-plane normal stress (σ_11_), shear stress (σ_13_), and *R*^2^ values with respect to the HZO deposition temperatures, 1D line plots for the HZO deposition temperatures of 200–300 °C extracted from GIWAXS results with Ψ increased in 5° steps from 0° to 90°, *P*–*E* curves of MFM capacitors with HZO deposition temperature of 200–300 °C, *P*–*E* curves of the HZO film deposited at 300 °C in its pristine state, remanent polarization extracted from the *P*–*E* curves at various triangular pulse frequencies of 1–10 kHz, *J*–*E* curves obtained from transient *I*–*V* measurements using triangular pulses at frequencies of 1, 2, 5, and 10 kHz, calculated ε_r_–*E* curves for the MFM capacitors with HZO deposition temperatures of 200–300 °C, combined ε_r_–*E* curves for the MFM capacitors with HZO deposition temperatures of 200–300 °C, normalized polarization as a function of the cumulative number of cycles for continuous cycling with a bipolar rectangular waveform at a frequency of 50 kHz and an amplitude of 3.0 MV/cm (PDF)
