# Supplementary material for: Temperature-Dependent {111}-Texture Transfer to Hf0.5Zr0.5O2 Films from {111}-Textured TiN Electrode and Its Impact on Ferroelectricity
Source: ACS Appl Mater Interfaces. 2025 Mar 28;17(18):26836–44. doi: 10.1021/acsami.4c17978 (PMC12067369; doi:10.1021/acsami.4c17978)
Supplement: Supplementary file 1 — am4c17978_si_001.pdf [file am4c17978_si_001.pdf]

Supporting information

Temperature-dependent {111}-Texture Transfer to $Hf_{0.5}Zr_{0.5}O_2$ Films from {111}-Textured TiN Electrode and Its Impact on Ferroelectricity

Dong Hee Han[1], Seung Yeon Kim[2], Hyun Woo Jeong[1], Younghwan Lee[3], Young Yong Kim[4,], Woojin Jeon[2,*], and Min Hyuk Park[1,5,6,*]*

[1]Department of Materials Science and Engineering & Inter-University Semiconductor Research Center, College of Engineering, Seoul National University, Seoul 08826, Republic of Korea

[2]Department of Advanced Materials Engineering for Information and Electronics, and Integrated Education Program for Frontier Science & Technology (BK21 Four), Kyung Hee University, Yongin, Gyeonggi, 17104, Korea

[3]School of Materials Science and Engineering, Chonnam National University, Gwangju, 61186, Republic of Korea

[4]Beamline Division, Pohang Accelerator Laboratory, POSTECH, Pohang 37673, Republic of Korea

[5]Research Institute of Advanced Materials, Seoul National University, Seoul 08826, Republic of Korea

[6]Institute of Engineering Research, Seoul National University, Seoul 08826, Republic of Korea

E-mail: Young Yong Kim (kimyy@postech.ac.kr), Woojin Jeon (woojin.jeon@khu.ac.kr), Min Hyuk Park (minhyuk.park@snu.ac.kr)

Figure S1a shows a schematic of the XRD method that used the Bragg–Brentano geometry. This geometry was used to measure the TiN thin film and confirm its preferred orientation. In the Bragg–Brentano geometry, the X-ray source and detector rise at the same angle, which ensures that the scattering vector is always normal to the surface, detecting diffractions only from planes parallel to the surface. Figure S1b shows the XRD pattern of TiN with a random orientation, whereas Figure S1c presents the XRD pattern of TiN with the {111} preferred orientation. As confirmed by the PDF, the powder diffraction of the randomly oriented TiN showed an intensity ratio of 72:100 between the (111) and (002) diffraction factors. In contrast, for the {111}-oriented TiN, the surface-normal diffraction of (002) ($2\theta \sim 42.6°$) was suppressed, and only the {111} diffraction ($2\theta \sim 36.7°$) was parallel to the surface. To control the preferred orientation of the TiN, we adjusted the Ar and N_2 gas flow ratio during the DC sputtering process. For the {111}-oriented TiN, an Ar:N_2 flow ratio of 20:5 in standard cubic centimeters per minute was used, whereas for the randomly oriented TiN, an Ar:N_2 flow ratio of 10:10 was employed. Controlling the preferred orientation of the TiN thin film in this manner played a critical role in

influencing the preferred orientation of the subsequent HZO thin film.[1] The resistivities of the deposited TiN films were measured using a four-point probe, with values of 586 ± 18 and 430 ± 15 μΩ·cm for the (111)-oriented and randomly oriented TiN films, respectively.

Greene *et al.*[2] reported differences in the preferred orientation of TiN films grown via the DC reactive sputtering process when adjusting the N_2 and Ti ion fluxes using an external magnetic field while keeping the ion irradiation energy at the substrate constant. When the Ti ion flux was high, {111}-oriented TiN formed, whereas an increase in the N_2^+ ion flux resulted in the formation of {002}-oriented TiN. In this study, the process used to fabricate {111}-oriented TiN involved increasing the Ar gas flow ratio, which in turn increased the relative Ti ion flux and reduced the N_2^+ ion flux. This trend was analogous to that reported by Greene *et al.*

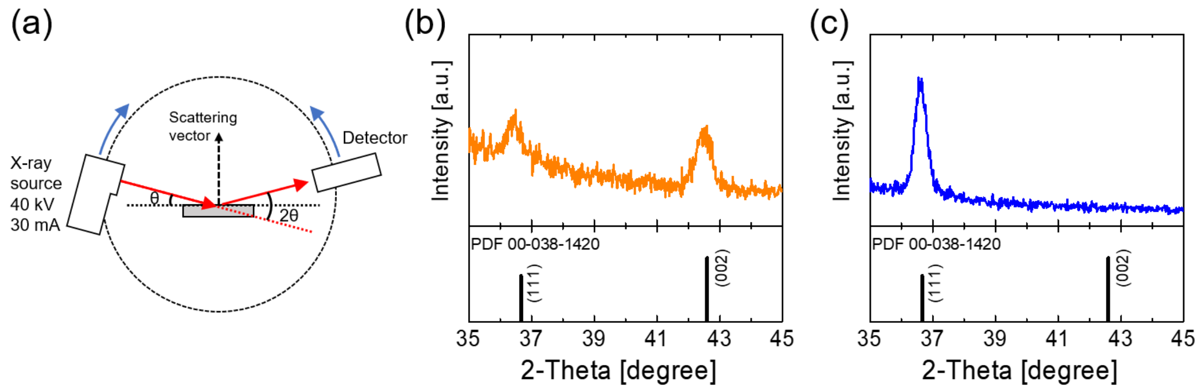

Figure S1. (a) X-ray diffraction (XRD) scheme with Bragg–Brentano geometry. XRD patterns of (b) random-oriented TiN film and (c) {111}-oriented TiN.

Figure S2 shows the XRD pattern of the HZO/TiN stack measured using the Bragg–Brentano geometry with various annealing temperatures (400-700 ºC). Despite varying the deposition temperature of the HZO from 200 to 300 ºC and the annealing temperatures, all the HZO films exhibited surface-normal crystallinity oriented along the o-{111}/t-{101} direction. No diffractions from o-(002)/o-(020)/t-{110} or the monoclinic phases near ~35.5° were observed. It is noteworthy that structural factors of the o-{111}/t-{101} and the o-(002)/o-(020)/t-{110} had values of 100 and 12–20, respectively. The diffraction peak at 2θ of 33° originated from the Si (002) diffraction of the Si substrate. HZO films deposited at higher temperatures (280 and 300 ºC) preferentially promote the {111} texture even at lower annealing temperature of 400 ºC. These results suggest that deposition temperature plays a critical role in the initial texture and crystallinity of HZO films, even before high-temperature annealing is performed. The diffraction patterns of the HZO/TiN stacks annealed at 700 ºC, at which complete crystallization occurred, were compared as shown in figure S3g. As the deposition temperature of HZO increased, the intensity of the o-{111} peak gradually increased. This indicated that the crystallinity of HZO in the o-{111} direction improved at higher deposition temperatures, suggesting stronger texture formation.

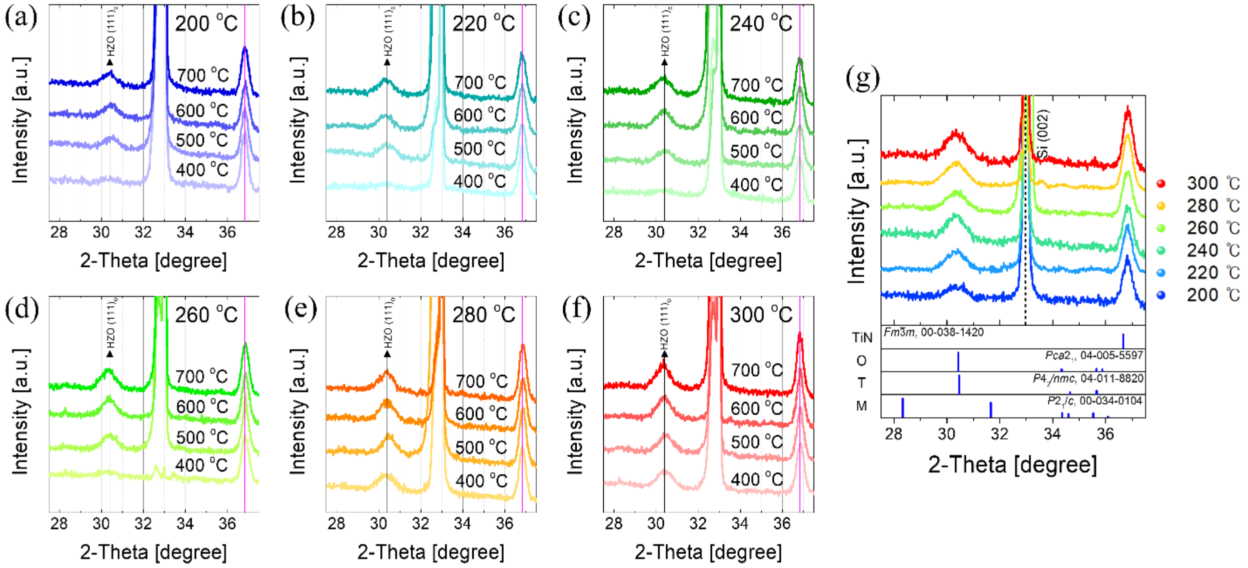

Figure S2. XRD patterns obtained using theta-2theta coupled geometry for the HZO/TiN stacks annealed at 400-700 ºC with HZO deposition temperatures of (a) 200 ºC, (b) 220 ºC, (c) 240 ºC, (d) 260 ºC, (e) 280 ºC, and (f) 300 ºC, respectively. The magenta line represents the TiN (111) diffraction. (g) XRD patterns obtained using Bragg-Brentano geometry, of HZO/TiN stacks at deposition temperatures of 200–300 °C after 700 ºC annealing.

Figure S3 presents the grazing-incidence X-ray diffraction (GIXRD) patterns of the HZO/TiN stacks, which were measured at an incidence angle of 0.5º. Across all the deposition temperatures for HZO, the most prominent diffraction peak was observed at a 2θ angle of 30.5º, corresponding to the o-{111}/t-{101} diffraction, with additional peaks from the monoclinic m-(-111) plane near 2θ = 28.5º and from the o-(002)/o-(020)/t-{110} plane near 2θ = 35.5º. Unlike in Figure S2, where only the surface-normal {111} plane is detected, the various diffractions in Figure S3 result from the fixed incidence angle (ω) used in the GIXRD geometry, while the detector (2θ) scanned over different angles. This caused the scattering vector to tilt as 2θ

increased, rather than remaining in the surface-normal direction in the Bragg–Brentano geometry. At a low annealing temperature of 400 ºC, the low o-{111} peak intensity indicates poor crystallinity due to insufficient annealing. It is observed that crystallization is completed at annealing temperatures of 600 ºC or higher for all HZO deposition temperatures. Therefore, the GIXRD patterns of HZO/TiN stacks annealed at 700 ºC were compared, as plotted in Figure S3g. As the deposition temperature increased, the intensity of the m-(-111) diffraction increased, whereas the intensity of the o-(002)/o-(020)/t-{110} diffraction decreased.

The m-phase is the most thermodynamically stable phase among the polymorphs of HfO_2 and ZrO_2, exhibiting the lowest Gibbs free energy, which allowed nucleation to occur during the deposition process. However, because of the high nucleation energy barrier, the t-phase typically crystallizes preferentially.[3] At higher deposition temperatures, atomic mobility increases, causing some t-phase nuclei in HZO films to transition into the m-phase, which results in the presence of more m-phase nuclei. These nuclei, which are formed under high-temperature deposition conditions, may exhibit higher crystallinity after crystallization. Similarly, the decreasing intensity of the o-(002)/o-(020)/t-{110} diffraction with increasing deposition temperature is attributed to the formation of more nuclei along the o-{111}/t-{101} plane, which has the lowest surface energy. This leads to preferential growth along the {111} plane and, in turn, suppresses growth along the o-(002)/o-(020)/t-{110} plane.

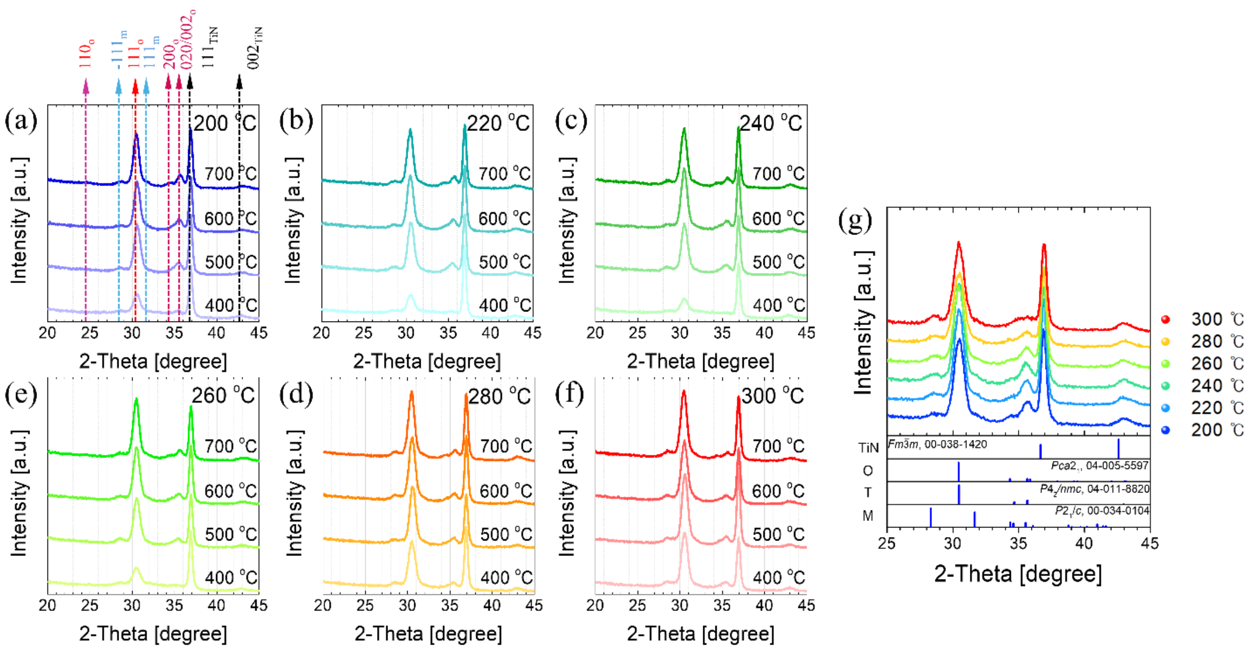

Figure S3. XRD patterns obtained using glazing incidence geometry with omega angle of 0.5º for the HZO/TiN stacks annealed at 400-700 ºC with HZO deposition temperatures of (a) 200 ºC, (b) 220 ºC, (c) 240 ºC, (d) 260 ºC, (e) 280 ºC, and (f) 300 ºC, respectively. (g) GIXRD patterns of HZO/TiN stacks after 700 ºC annealing with the HZO deposition temperatures of 200–300 ºC.

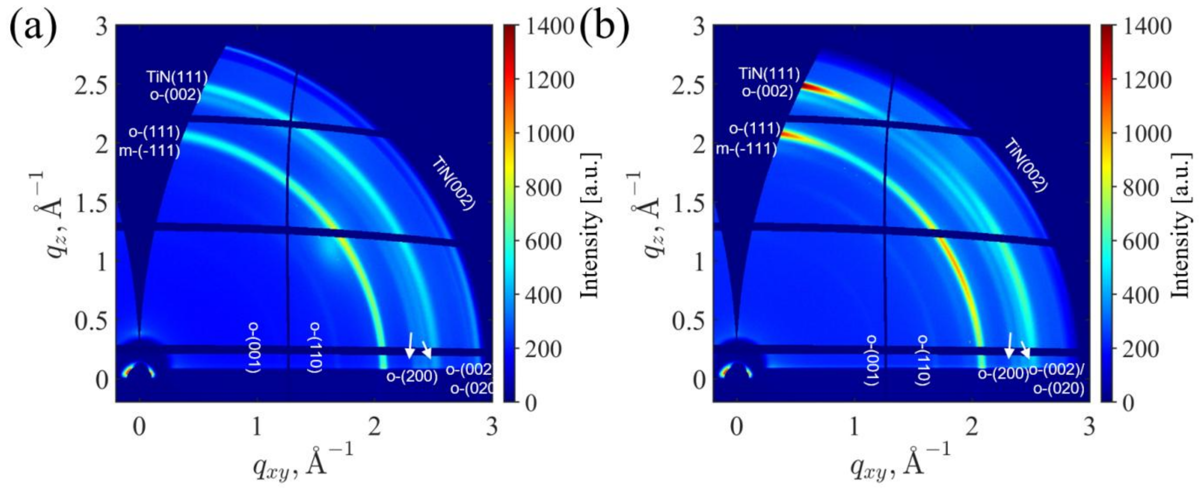

Figure S4. GIWAXS 2D images of (a) HZO film with randomly oriented TiN and (b) HZO film with a (111)-oriented TiN, where the HZO deposition temperature was 240 ºC.

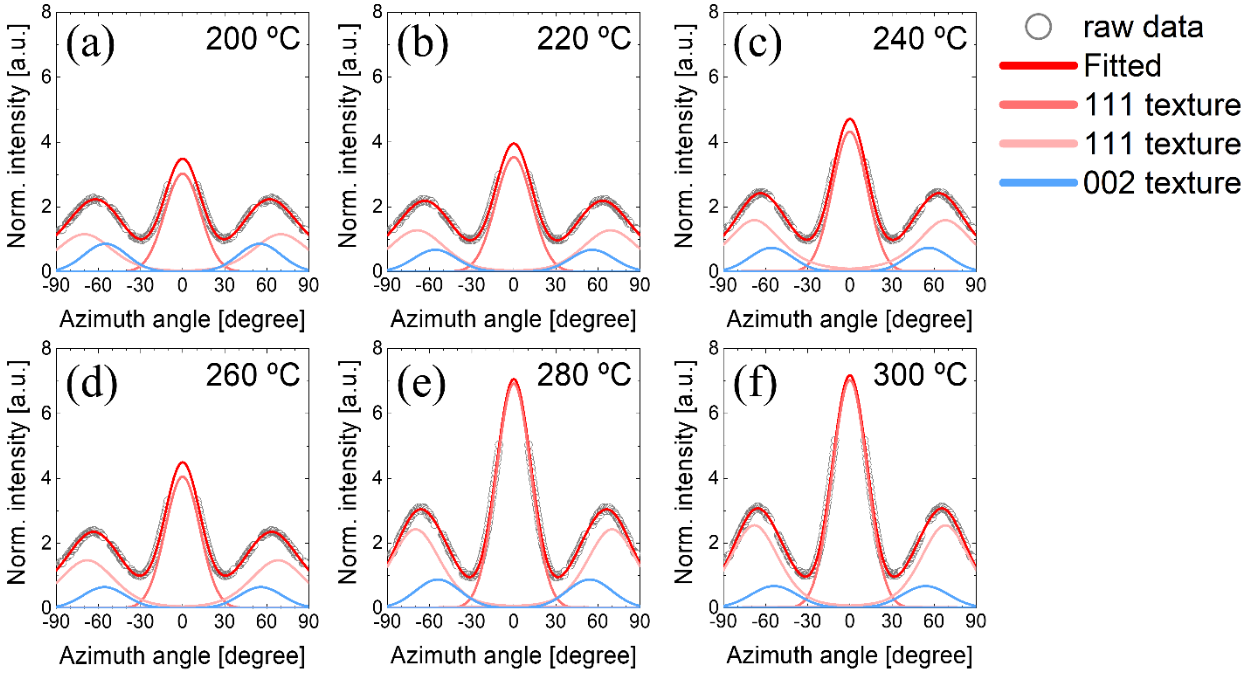

Figure S5. Normalized intensity plots according to azimuth angle at q ~ 2.11 Å^{-1} of o-{111} for HZO deposition temperatures of (a) 200, (b) 220, (c) 240, (d) 260, (e) 280, and (f) 300 ºC.

	Out-of-plane {111} texture			{111} texture			{002} texture		
Deposition temperature [ºC]	Scale	Mean	FWHM	Scale	Mean	FWHM	Scale	Mean	FWHM
200	96.70	0	29.97	64.86	70.00	45.99	33.18	54.95	36.00
220	114.03	0	30.33	69.71	68.94	45.99	25.88	55.99	36.00
240	133.47	0	29.00	99.08	68.00	46.00	28.36	56.00	36.00
260	125.43	0	29.03	84.32	68.00	46.00	25.00	55.74	36.00
280	213.76	0	29.00	125.33	70.00	41.90	35.17	54.05	37.43
300	216.90	0	29.00	129.34	68.00	41.08	27.93	54.00	38.87

Table S1. Fitting parameters of o-{111} azimuthal analysis, where fitting was conducted using a pseudo-Voigt distribution function.

Deposition temperature [ºC]	**Out-of-plane {111} texture**	**{111} texture**	**{002} texture**
200	0.54	0.28	0.18
220	0.58	0.29	0.13
240	0.57	0.32	0.12
260	0.58	0.31	0.11
280	0.61	0.29	0.10
300	0.62	0.30	0.08

Table S2. Normalized integrated peak areal ratios of the deconvoluted peaks from Table S1.

Figure S6a presents the GIXRD patterns measured after the ALD of HZO on (111)-oriented TiN to examine the influence of the deposition temperature on nucleation. At all the deposition temperatures, the HZO films remained amorphous, as indicated by the broad humps observed in the diffraction patterns. However, the increase in the intensity of the hump with the deposition temperature suggests an increase in the possibility of nucleation. Figure S6b shows a Arrhenius-type plot of the integrated areal ratio of the hump normalized with respect to the TiN (111) peak at each deposition temperature. As the deposition temperature increased, the areal ratio decreased, indicating an increase in the possibility of nucleation.

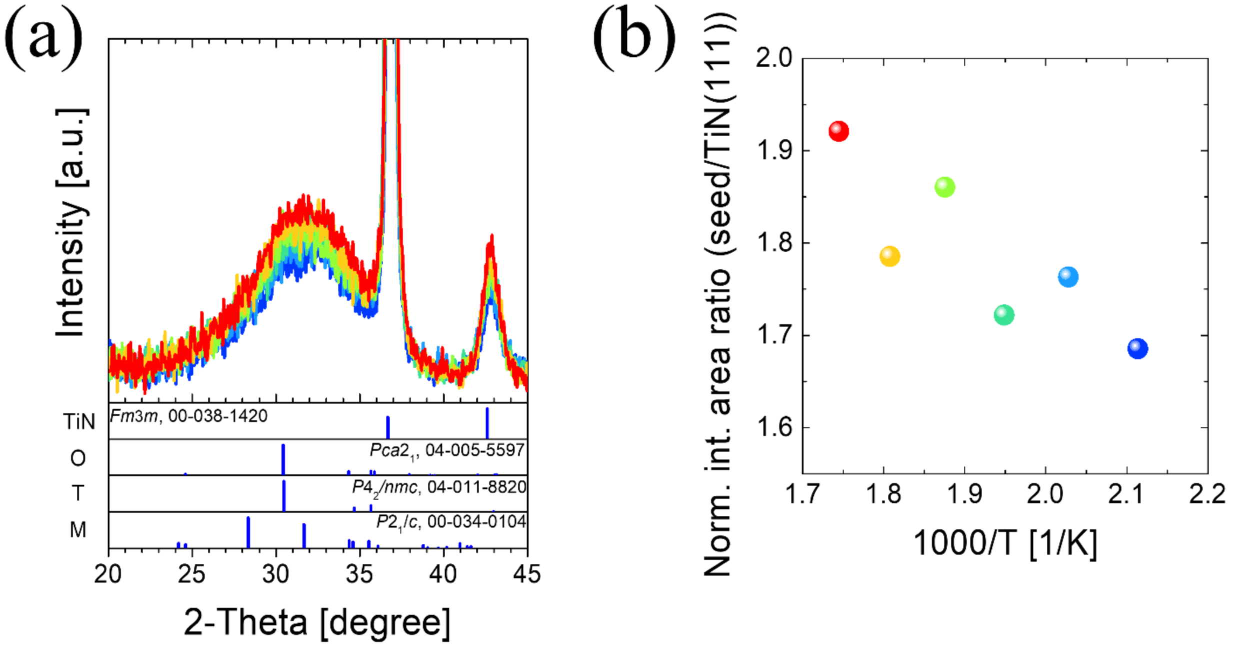

Figure S6. (a) GIXRD patterns of the same HZO/TiN stacks before crystallization at these deposition temperatures. (b) normalized intensity ratio of seed and TiN(111) diffractions.

Effect of ALD oxidant on {111}-texture transfer between TiN and HZO films

We deposited HZO thin films on a (111)-textured TiN bottom electrode using O_3 or H_2O as oxidants and compared their crystal structures using XRD. As shown in Figure S7, the use of O_3 as an oxidant weakened the {111}-texture of the HZO, and this tendency became more pronounced as the deposition temperature increased from 200 to 300 ºC. This indicates that a stronger oxidant promotes the oxidation of the TiN electrode, thereby weakening the {111}-texture transfer from TiN to HZO.

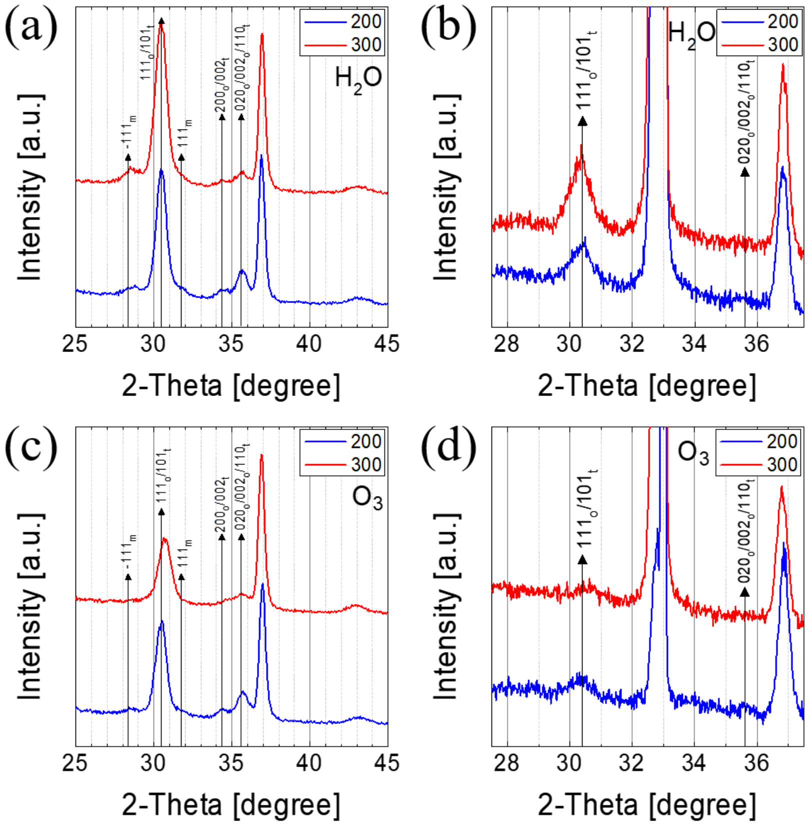

Figure S7. (a) and (c) show the GIXRD patterns of the HZO/TiN stacks, where H_2O and O_3 were used as reactants, respectively. (b) and (d) display the XRD patterns with a theta-2theta coupled geometry for the HZO/TiN stacks. The blue and red lines represent the HZO deposition temperatures of 200 ºC and 300 ºC, respectively.

Derivation of the equation for stress analysis

A method for analyzing the residual stress in textured thin films using the GIXRD geometry was reported by Ma *et al.*[6]

Because the stress in the films was biaxial, the following stress tensor could be used:

$$\begin{pmatrix} \sigma_{11} & 0 & 0 \\ 0 & \sigma_{11} & 0 \\ 0 & 0 & 0 \end{pmatrix}. \quad (1)$$

Because strong non-linearity could be observed from the d-spacing - $\cos^2\alpha\sin^2\Psi$ plot, assuming the existence of shear stress in the out-of-plane direction, the stress tensor can be defined as follows:

$$\begin{pmatrix} \sigma_{11} & 0 & \sigma_{13} \\ 0 & \sigma_{11} & \sigma_{13} \\ \sigma_{13} & \sigma_{13} & 0 \end{pmatrix}. \quad (2)$$

In Figures S8c and d, S_i (i = 1, 2, and 3) represents the axes of sample system **S**, while L_i (i = 1, 2, and 3) represents the axes of laboratory system **L**. In the GIXRD geometry, the laboratory system is first rotated about the S_1 axis by an angle, Ψ, relative to the sample system. Then, it is rotated about the S_2 axis by angle α. Because the d-spacing of the (*hkl*) plane changes owing to the residual stress applied to the thin film, the strain on the (*hkl*) plane is defined as follows:

$$\varepsilon_{\alpha\Psi} = \varepsilon_{33}^{L} = \frac{d_{\alpha\Psi} - d_0}{d_0}, \quad (3)$$

where $d_{\alpha\Psi}$ and d_0 represent the d-spacing of the (*hkl*) plane when $\Psi \neq 0^{\circ}$ and $\Psi = 0^{\circ}$, respectively.

A rotational transformation process is required to explain the data measured in the laboratory system. The matrix for the tensor transformation is defined as follows:

$$\varepsilon_{33}^{L} = A_{3k} A_{3l} \varepsilon_{kl}^{S}, \quad (4)$$

$$A_{ik} = \begin{vmatrix} \cos\alpha & 0 & -\sin\alpha \\ \sin\alpha\sin\Psi & \cos\Psi & \cos\alpha\sin\Psi \\ \sin\alpha\cos\Psi & -\sin\Psi & \cos\alpha\cos\Psi \end{vmatrix}. \quad (5)$$

Using equations (3), (4), and (5), the following can be calculated.

$$\varepsilon_{33}^{L} = \sin^2\alpha\cos^2\Psi\,\varepsilon_{11} - \sin\alpha\sin 2\Psi\,\varepsilon_{12} + \sin 2\alpha\cos^2\Psi\,\varepsilon_{13} + \sin^2\Psi\,\varepsilon_{22} -$$

$$\sin 2\Psi\cos\alpha\,\varepsilon_{23} + \cos^2\alpha\cos^2\Psi\,\varepsilon_{33} \quad (6)$$

In the case of isotropic materials, the strain can be expressed as follows:

$$\varepsilon_{ij} = \frac{1+\nu}{E}\sigma_{ij} - \delta_{ij}\frac{\nu}{E}\sigma_{kk}, \quad (7)$$

where ν and E represent Poisson's ratio and Young's modulus, respectively.

Substituting the above equation into equation (6), the following is derived:

$$\varepsilon_{33}^{L} = \frac{1+\nu}{E}(\sigma_{22} - \sigma_{11}\sin^2\alpha - \sigma_{33}\cos^2\alpha)\sin^2\Psi + \frac{1+\nu}{E}(\sigma_{11}\sin^2\alpha + \sigma_{33}\cos^2\alpha) -$$

$$\frac{\nu}{E}(\sigma_{11} + \sigma_{22} + \sigma_{33}) - \frac{1+\nu}{E}(\sigma_{12}\sin\alpha\sin 2\Psi + \sigma_{23}\cos\alpha\sin 2\Psi - \sigma_{13}\sin 2\alpha\cos^2\Psi). \quad (8)$$

Using the previously assumed equation (2), equation (8) can be reduced to the following:

$$d_{\alpha\psi} = \frac{1+\nu}{E} d_0\sigma_{11}\, cos^2\,\alpha\, sin^2\,\psi + \frac{1+\nu}{E} d_0\sigma_{11}\, sin^2\,\alpha - \frac{2\nu}{E} d_0\sigma_{11} + d_0 -$$

$$\frac{1+\nu}{E} d_0\sigma_{13}\,(cos\,\alpha\, sin\, 2\psi - sin\, 2\alpha\, cos^2\,\psi). \quad (9)$$

By applying a nonlinear fitting process to the d-spacing and Ψ values using equation (9), σ_{11} and σ_{13} can be obtained.

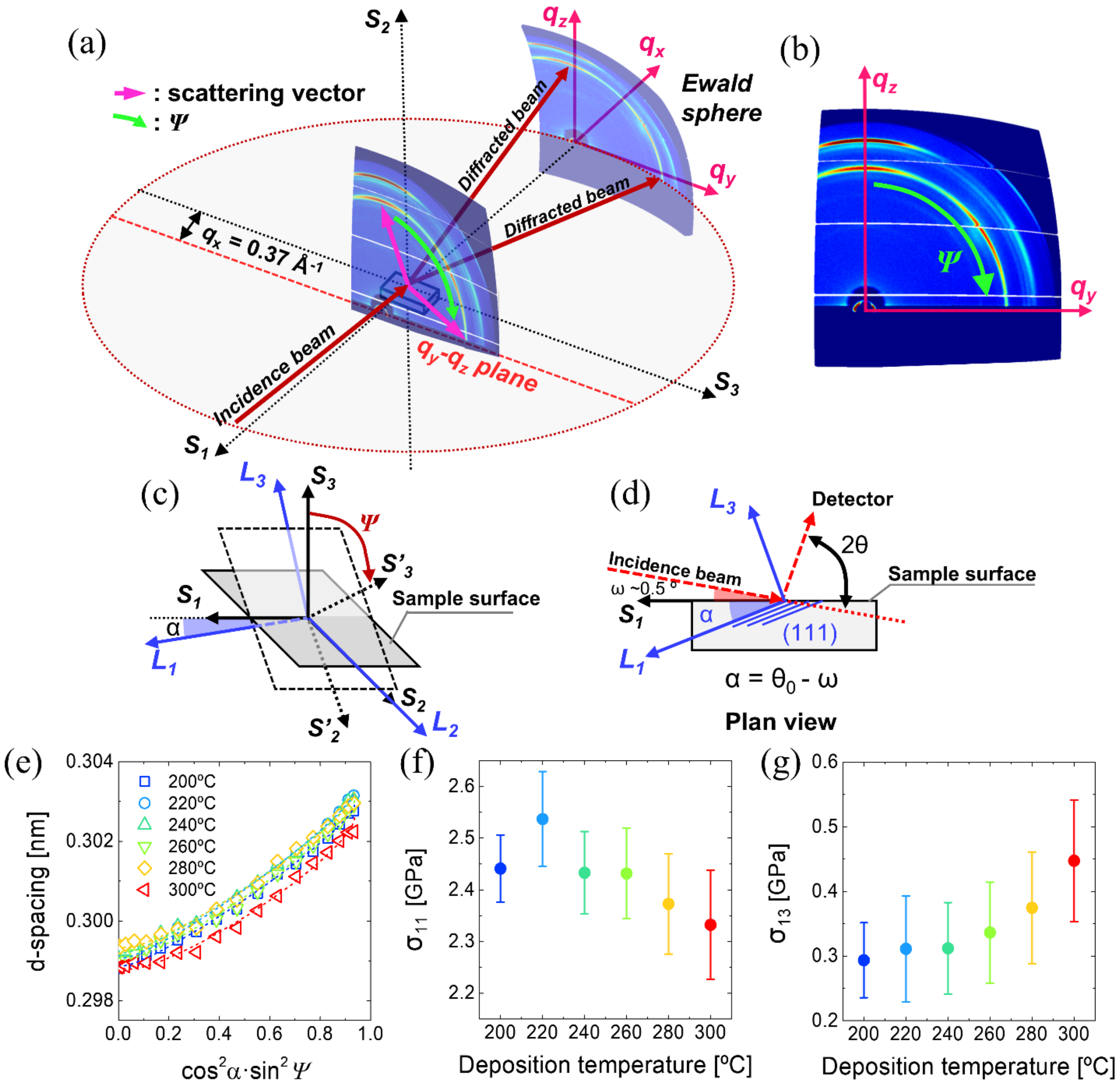

Figure S8. (a) Schematic illustration showing the q_{xy}-q_z image on the surface of the Ewald sphere and its projection onto the q_y-q_z plane, neglecting the q_x component. The geometry accounts for the Ewald sphere effect, with the q_x component of the o-{111} diffraction assumed to be constant at q_x = 0.37 Å^{-1}, resulting in the q_y-q_z image lying on the plane intersecting the

Ewald sphere. (b) q_y-q_z GIWAXS image, indicating angle Ψ. (c) Schematic of laboratory coordinate system L_i, sample coordinate system S_i, and angles α and Ψ. (d) Plan view of laboratory coordinates, incidence X-ray and diffracted beams, and angle α. (e) Lattice distance (d-spacing) versus $cos^2\alpha sin^2\Psi$ plot of the HZO films. Deposition temperature-dependent (f) normal stress σ_{11} and (g) shear stress σ_{13}.

Stress analysis results using modified $\sin^2\Psi$ method with GIWAXS measurements

To examine the effect of the deposition temperature on the mechanical stress evolution in the HZO films during the texture transfer, a modified version of the $\sin^2\Psi$ analysis was conducted based on the GIWAXS results, with a schematic of the geometry and q_y-q_z plane GIWAXS image shown in Figures S8a and b, respectively. Figure S8a shows the geometry used as the basis for the modified stress analysis. Axes S_1, S_2, and S_3 represent the principal directions of the measured samples. A q_{xy}-q_z diffraction image of the surface of the Ewald sphere was obtained. It was assumed that the x-component of the o-{111} diffraction in q-space (q_x) remained constant at 0.37 $Å^{-1}$. Consequently, a new GIWAXS image was constructed on the q_y-q_z plane at a distance of 0.37 $Å^{-1}$ from the origin. In this configuration, the angle between the diffraction and q_z, as viewed from the origin, was defined as the psi angle (Ψ) in the stress analysis. Figure S8b shows the aforementioned q_y-q_z GIWAXS plane image and a definition of Ψ. Figure S8e shows the d-spacing versus $\cos^2\alpha\sin^2\Psi$ plot based on changes in the o-{111} peak position, which were dependent on Ψ. One noticeable point is that rather strong non-linearity could be observed from the d-spacing versus $\cos^2\alpha\sin^2\Psi$ plot, which has been known to originate from factors such as a strain-gradient and the existence of shear strain. Because of the fixed incidence angle for the GIWAXS geometry, there was no change in the penetration depth dependent on Ψ. Thus, the

shear stress was considered a potential reason for the observed non-ideal non-linearity. The large deviation observed at Ψ = 45° also quantitatively supported the hypothesis of the shear stress effect, because the magnitude of the shear stress effect was proportional to the additional term.[7]

A method for analyzing residual stress in textured thin films using the GIXRD geometry was reported by Ma *et al.*[6] The key difference from the well-known $\sin^2\Psi$ method is the introduction of $\cos^2\alpha$ to incorporate a geometric correction factor, where α is the difference between Bragg diffraction angle θ and glancing angle ω, as shown in Figures S8c and d. The residual shear stress component was also considered to analyze the unexpected nonlinearity in the films. Using this approach, we extracted the o-{111} peak position from the GIWAXS 1D line plot to calculate the residual stress in the HZO film using equation (10), which considers both the normal and shear stress components. The details of the derivation of equation (10) can be found as mentioned above.

$$d_{\alpha\psi} = \frac{1+\nu}{E} d_0\sigma_{11} \cos^2\alpha \sin^2\psi + \frac{1+\nu}{E} d_0\sigma_{11} \sin^2\alpha - \frac{2\nu}{E} d_0\sigma_{11} + d_0 - \frac{1+\nu}{E} d_0\sigma_{13} (\cos\alpha \sin 2\psi - \sin 2\alpha \cos^2\psi) \quad (10)$$

Here, d_0 is the initial lattice spacing when Ψ = 0, $d_{\alpha\Psi}$ is the lattice spacing with a specific Ψ, E is Young's modulus, ν is Poisson's ratio, σ_{11} is the residual normal stress, σ_{13} is the shear stress, and α is equal to a referenced Bragg angle of the observed plane (hkl) minus a glancing angle of 0.5°.

Figure S8f presents the residual in-plane normal stress ($\sigma_{11}= \sigma_{22}$) of the HZO o-{111} plane as a function of the deposition temperature, while Figure S8g shows the residual shear stress ($\sigma_{13}= \sigma_{23}$) calculated using Poisson's ratio (ν = 0.29) and Young's modulus (E = 209 GPa) from the

literature.[8-12] The magnitudes of the in-plane normal and shear stresses are included in Table S3, while the 1D line plots of the GIWAXS results used for the stress analysis are included in Figure S9. Figures S8f and g show that the residual in-plane normal stress decreased by 4.5% (from 2.44 to 2.33 GPa) and residual shear stress increased by 55.2% (from 0.29 to 0.45 GPa), respectively, with an increase in the deposition temperature from 200 to 300 ºC. From these results, it is believed that with the strengthening of the {111} texture from the {111} TiN electrode to the HZO thin films, the residual in-plane normal stress was released, while the shear stress increased. These results indicate that as the HZO films develop a stronger {111} texture, which is known to enhance total ferroelectricity compared to the randomly-oriented film, the residual shear stress is observed to increase significantly. The strengthening of the {111} texture not only aligns the crystallographic structure but also introduces higher shear stress within the film.

Although the exact origin of the observed shear stress has yet to be elucidated, some hypotheses can be proposed based on the observations of the structural analysis in this study. As shown in the GIXRD patterns of the as-deposited HZO films deposited at various temperatures from 200 to 300 °C in Figure S4a, the location of the diffraction peak was consistent with that of the m-{111} diffraction peak, suggesting that the crystallographic structure of the nano-crystallites in the as-deposited films would be the m-phase. However, after the crystallization using rapid thermal process (RTP), the dominant crystallographic phase was the ferroelectric o-phase, which suggested that there was a transition from the m-phase to o-phase, as reported by Mimura *et al.*[13] in the epitaxial Y-doped HfO_2 thin films. The intermediate transition phase at elevated temperatures during RTP would have been the t-phase, as suggested in previous studies,[13-14] although there was no direct evidence of the t-phase transition in this study. It is known that the

transition between the polymorphs of HfO_2 or ZrO_2 is based on the martensitic transformation,[15-16] which affects the shear stress in thin films.

	200 ºC	220 ºC	240 ºC	260 ºC	280 ºC	300 ºC
σ_{11} [GPa]	2.44 ± 0.08	2.54 ± 0.12	2.43 ± 0.10	2.43 ± 0.11	2.37 ± 0.12	2.33 ± 0.13
σ_{13} [GPa]	0.29 ± 0.06	0.31 ± 0.08	0.31 ± 0.07	0.34 ± 0.08	0.37 ± 0.09	0.45 ± 0.09
R^2	0.998	0.996	0.997	0.996	0.995	0.994

Table S3. Magnitudes of the in-plane normal stress (σ_{11}), shear stress (σ_{11}), and R^2 values with respect to the HZO deposition temperatures.

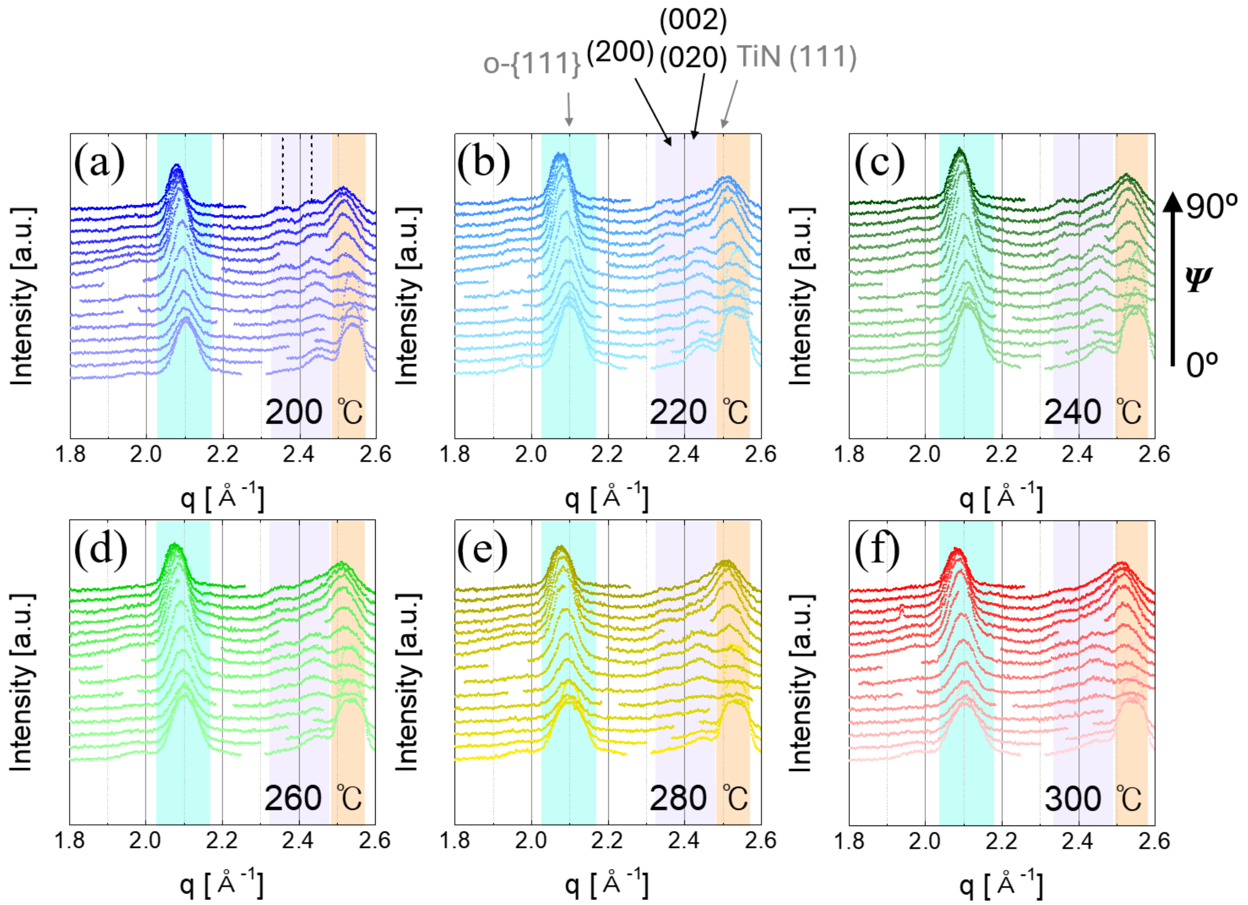

Figure S9. 1D line plots for the HZO deposition temperatures of (a) 200, (b) 220, (c) 240, (d) 260, (e) 280, and (f) 300 ºC extracted from GIWAXS results with Ψ increased in 5º steps from 0º to 90º.

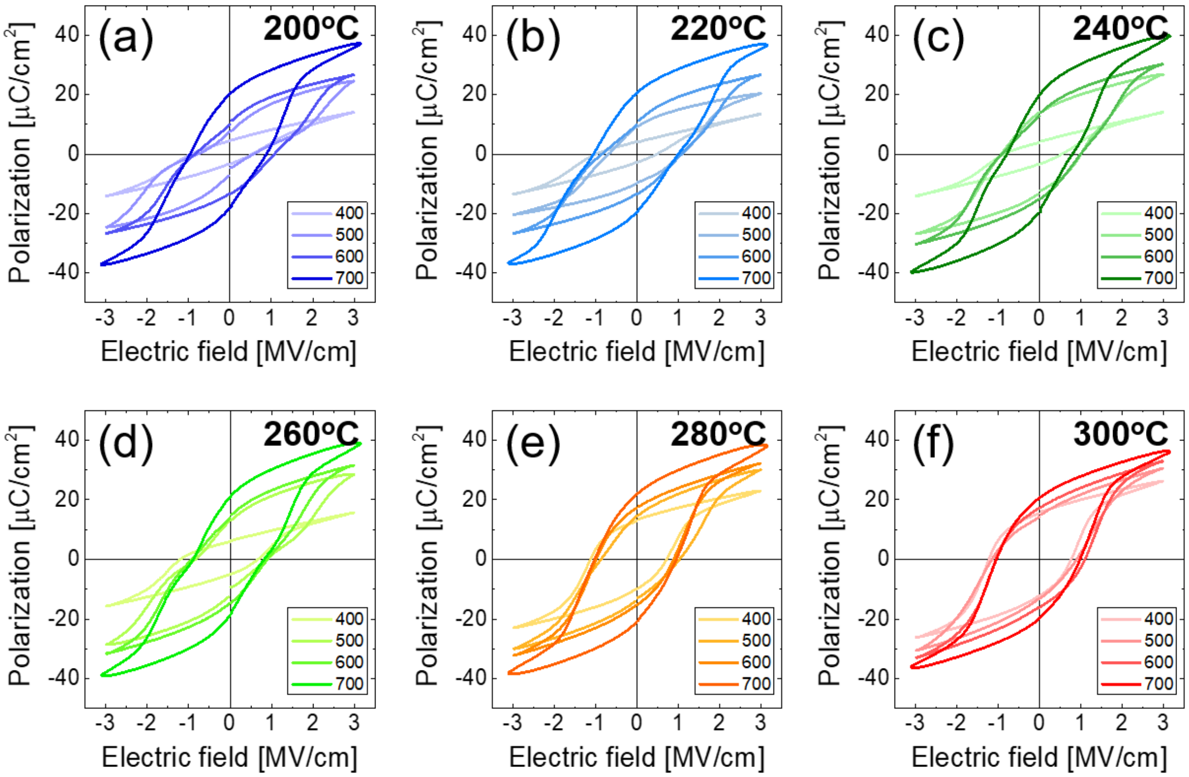

Figure S10. P-E curves of MFM capacitors with HZO deposition temperature of (a) 200 ºC, (b) 220 ºC, (c) 240 ºC, (d) 260 ºC, (e) 280 ºC, and (f) 300 ºC, respectively. The legend indicates annealing temperatures.

Frequency-dependent P-E curves

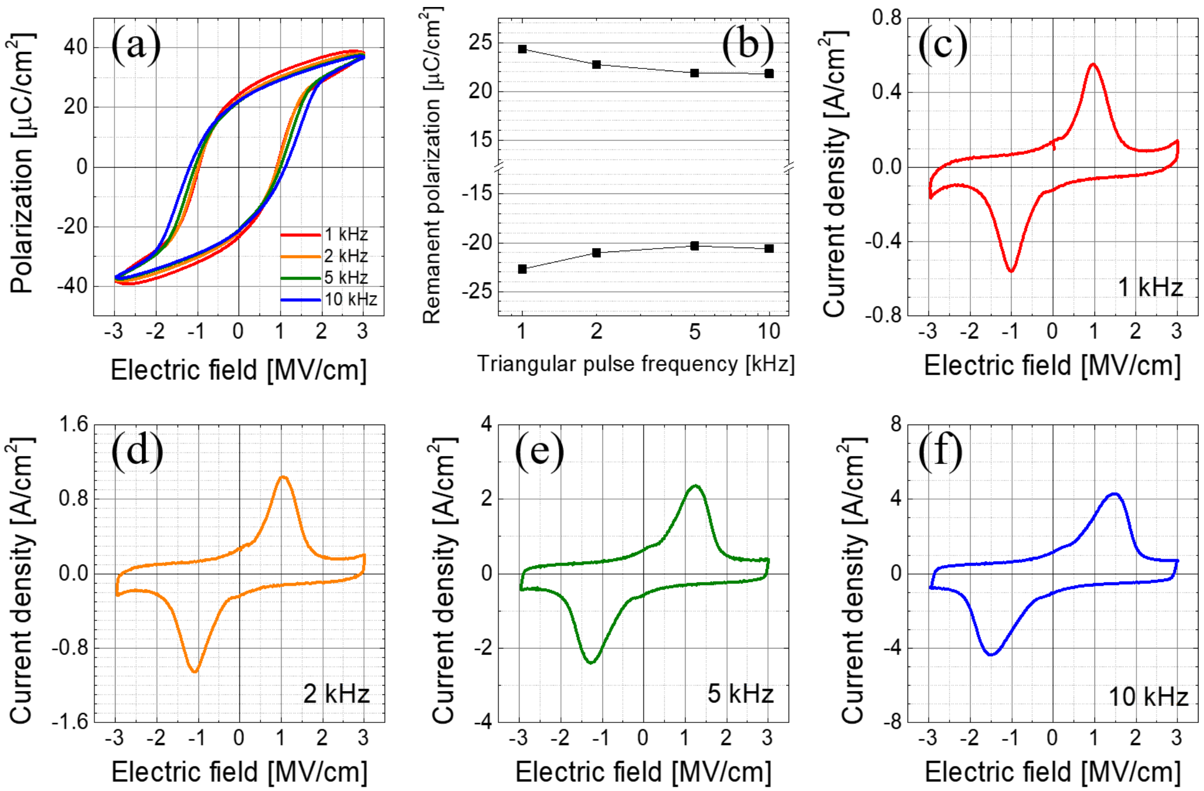

Figure S11. (a) P-E curves of the HZO film deposited at 300 ºC in its pristine state. (b) Remanent polarization extracted from the P-E curves at various triangular pulse frequencies of 1, 2, 5, and 10 kHz. (c), (d), (e), and (f) show the J-E curves obtained from transient I-V measurements using triangular pulses at frequencies of 1, 2, 5, and 10 kHz, respectively.

Figure S11 shows frequency-dependent P-E curve measured by using 1, 2, 5, 10 kHz triangular bipolar pulses. As the pulse frequency increases, the leakage current component at high *E* decreases as shown in *J-E* curves of the figure S11c-f. This reduction is due to the limited time available for charge migration through leakage pathways at higher frequencies. Consequently, the remanent polarization also decreases with increasing frequency.

Higher frequencies lead to an increase in the coercive field. This frequency dependence of coercive field (E_c) can be attributed to both intrinsic and extrinsic factors. Intrinsically, domain nucleation and growth mechanisms contribute to an increase in E_c at higher frequencies, as switching kinetics are constrained by the limited time available for domain wall motion. Extrinsically, interfacial passive layers between the film and electrodes, as well as contact resistance at the electrode interfaces, can introduce additional voltage drops, leading to an overestimation of E_c in P-E hysteresis loop measurements. This effect has been previously discussed in studies on domain switching time and interfacial resistance effects on ferroelectric thin films.[4-5]

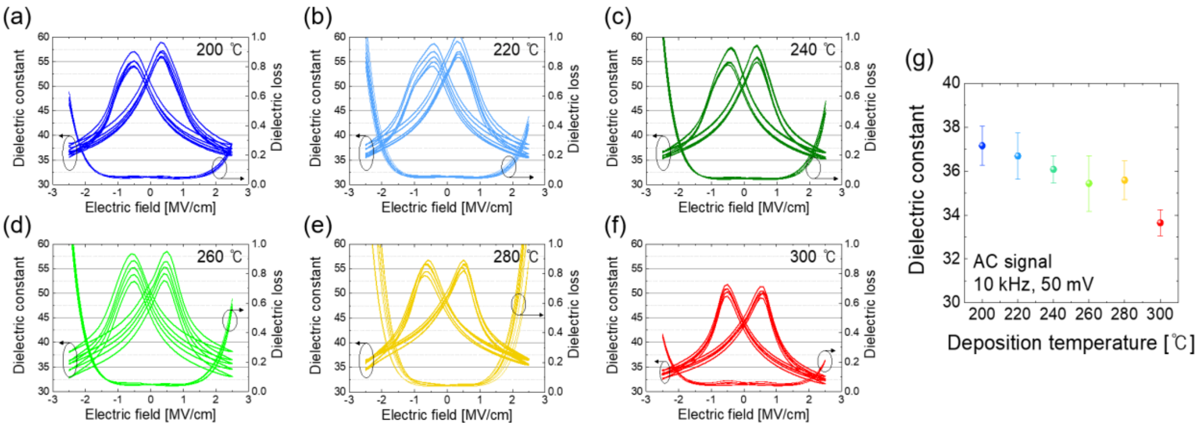

Figure S4. Calculated εr-E curves for the MFM capacitors with HZO deposition temperatures of (a) 200, (b) 220, (c) 240, (d) 260, (e) 280, and (f) 300 ºC. (g) Combined εr-E curves for the MFM capacitors at all deposition temperatures. The frequency and amplitude of the AC small signal were 10 kHz and 50 mV, respectively

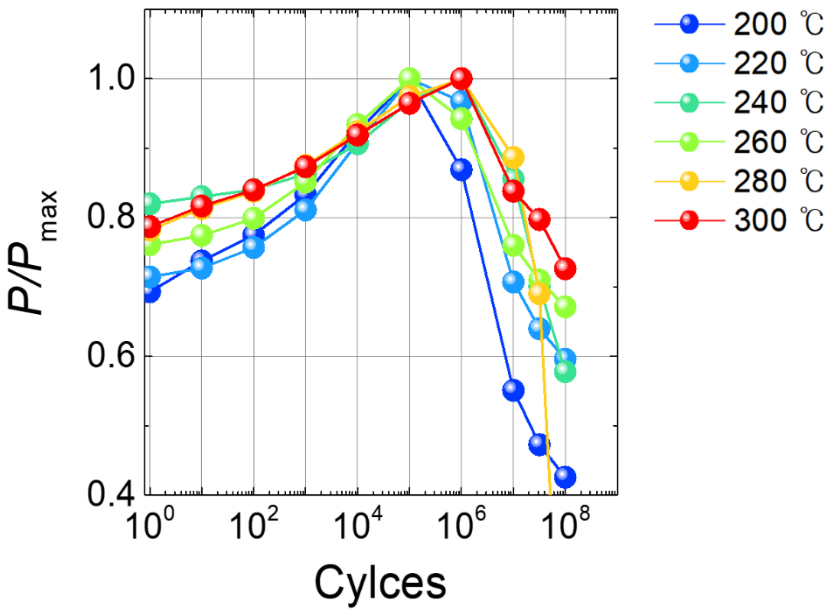

Figure S5. Normalized polarization as a function of the cumulative number of cycles for continuous cycling with a bipolar rectangular waveform at a frequency of 50 kHz and an amplitude of 3.0 MV/cm.

To analyze the factors contributing to the orientational rotation between the (200)-, (020)-, and (002)-oriented domains, which were associated with the wake-up effect, the intensities extracted from the Debye–Scherrer ring of the HZO/TiN stack at deposition temperatures of 200 and 300 ºC are shown in Figure S9. The intensities were extracted at 5° increments of Ψ from 0º to 90º and plotted as a one-dimensional line. At a lower deposition temperature of 200 ºC, crystallinity in the in-plane directions of (200) and (020) (including (002)) was prominently observed, whereas at the higher deposition temperatures, the crystallinity of these planes was less pronounced. Considering that the symmetry of (200), (020), and (002) appears at 90º intervals, it can be inferred that the HZO film deposited at 200 °C showed strong orientation in the (111) direction, while also exhibiting a preferred orientation along (200)/(020)/(002).

It should be noted that the (020) and (002) planes in HfO_2 $Pca2_1$ (with lattice parameters of a: 5.22 Å, b: 5.00 Å, and c: 5.03 Å) reflect at 35.856º and 35.65º under Cu Kα radiation, making them difficult to distinguish. The strong (200) and (020) diffractions in the in-plane direction provide structural conditions conducive to rotational orientation evolution, contributing to changes in the domain structure and leading to a wake-up effect where polarization increases during early cycling. In contrast, the HZO film deposited at the higher temperature of 300 °C exhibited a much stronger orientation in the (111) direction, with significantly reduced intensities for the (200), (020), and (002) planes in the in-plane direction. This can be interpreted as the result of the numerous nuclei formed during the high-temperature deposition process crystallizing in the energetically stable {111} direction after grain growth, which suppressed the orientation of the other crystal planes. The {111} plane was oriented perpendicular to the electric field direction, which reduced the potential for rotational orientation evolution.

REFERENCES

(1) Lee, Y.; Broughton, R. A.; Hsain, H. A.; Song, S. K.; Edgington, P. G.; Horgan, M. D.; Dowden, A.; Bednar, A.; Lee, D. H.; Parsons, G. N.; et al. The Influence of Crystallographic Texture on Structural and Electrical Properties in Ferroelectric $Hf_{0.5}Zr_{0.5}O_2$. *Journal of Applied Physics* **2022**, *132,* 244103. DOI: 10.1063/5.0128038.

(2) Greene, J.; Sundgren, J. E.; Hultman, L.; Petrov, I.; Bergstrom, D. Development of Preferred Orientation in Polycrystalline TiN Layers Grown by Ultrahigh Vacuum Reactive Magnetron Sputtering. *Applied physics letters* **1995**, *67* (20), 2928-2930. DOI: 10.1063/1.114845.

(3) Ye, K. H.; Yeu, I. W.; Han, G.; Jeong, T.; Yoon, S.; Kim, D.; Hwang, C. S.; Choi, J.-H. Comprehensive Interpretations of Thermodynamic and Kinetic Effects on the Phase Fractions in $Hf_{1-x}Zr_xO_2$ by first principle calculations. *Applied Physics Reviews* **2023**, *10,* 031419. DOI: 10.1063/5.0160719

(4) Li, S.; Zhou, D.; Shi, Z.; Hoffmann, M.; Mikolajick, T.; Schroeder, U. Involvement of Unsaturated Switching in the Endurance Cycling of Si-Doped HfO_2 Ferroelectric Thin Films. *Adv. Electron. Mater.* **2020**, 6 (8), 2000264.

(5) Jiang, A.Q.; Zhang, D.W.; Estimation of film-electrode contact resistance and domain switching time from ferroelectric polarization-voltage hysteresis loops. *Thin Solid Films* 545 (2013) 145-148.

(6) Ma, C.-H.; Huang, J.-H.; Chen, H. Residual Stress Measurement in Textured Thin Film by Grazing-Incidence X-ray Diffraction. *Thin Solid Films* **2002**, *418* (2), 73-78. DOI: 10.1016/S0040-6090(02)00680-6

(7) Sarmast, A.; Schubnell, J.; Preußner, J.; Hinterstein, M.; Carl, E. Residual Stress Analysis in Industrial Parts: A Comprehensive Comparison of XRD Methods. *Journal of Materials Science* **2023**, *58* (44), 16905-16929. DOI: 10.1007/s10853-023-09069-z.

(8) Selcuk, A.; Atkinson, A. Elastic Properties of Ceramic Oxides Used in Solid Oxide Fuel Cells (SOFC). *Journal of the European Ceramic Society* **1997**, *17* (12), 1523-1532. DOI: 10.1016/S0955-2219(96)00247-6.

(9) Zhao, X.-S.; Shang, S.-L.; Liu, Z.-K.; Shen, J.-Y. Elastic Properties of Cubic, Tetragonal and Monoclinic ZrO_2 from First-Principles Calculations. *Journal of Nuclear Materials* **2011**, *415* (1), 13-17. DOI: 10.1016/j.jnucmat.2011.05.016.

(10) Dole, S.; Hunter Jr, O.; Wooge, C. Elastic properties of Monoclinic Hafnium Oxide at Room Temperature. *Journal of the American Ceramic Society* **1977**, *60* (11-12), 488-490. DOI: 10.1111/j.1151-2916.1977.tb14088.x.

(11) Fields, S. S.; Cai, T.; Jaszewski, S. T.; Salanova, A.; Mimura, T.; Heinrich, H. H.; Henry, M. D.; Kelley, K. P.; Sheldon, B. W.; Ihlefeld, J. F. Origin of Ferroelectric Phase Stabilization via the Clamping Effect in Ferroelectric Hafnium Zirconium Oxide Thin Films. *Advanced Electronic Materials* **2022**, *8* (12), 2200601. DOI: 10.1002/aelm.202200601.

(12) Fields, S. S.; Olson, D. H.; Jaszewski, S. T.; Fancher, C. M.; Smith, S. W.; Dickie, D. A.; Esteves, G.; Henry, M. D.; Davids, P. S.; Hopkins, P. E. Erratum:"Compositional and Phase

Dependence of Elastic Modulus of Crystalline and Amorphous $Hf_{1-x}Zr_xO_2$ Thin Films"[Appl. Phys. Lett. 118, 102901 (2021)]. *Applied Physics Letters* **2021**, *119,* 129901. DOI: 10.1063/5.0068886.

(13) Mimura, T.; Shimizu, T.; Kiguchi, T.; Akama, A.; Konno, T. J.; Katsuya, Y.; Sakata, O.; Funakubo, H. Effects of Heat Treatment and in situ High-Temperature X-ray Diffraction Study on the Formation of Ferroelectric Epitaxial Y-doped HfO_2 Film. *Japanese Journal of Applied Physics* **2019**, *58* (SB), SBBB09. DOI: 10.7567/1347-4065/aafed1.

(14) Schroeder, U.; Park, M. H.; Mikolajick, T.; Hwang, C. S. The Fundamentals and Applications of Ferroelectric HfO_2. *Nature Reviews Materials* **2022**, *7* (8), 653-669. DOI: 10.1038/s41578-022-00431-2.

(15) Tang, J.; Zhang, F.; Zoogman, P.; Fabbri, J.; Chan, S. W.; Zhu, Y.; Brus, L. E.; Steigerwald, M. L. Martensitic Phase Transformation of Isolated HfO_2, ZrO_2, and $Hf_xZr_{1-x}O_2$ ($0 < x < 1$) Nanocrystals. *Advanced Functional Materials* **2005**, *15* (10), 1595-1602. DOI: 10.1002/adfm.200500050.

(16) Hudak, B. M.; Depner, S. W.; Waetzig, G. R.; Talapatra, A.; Arroyave, R.; Banerjee, S.; Guiton, B. S. Real-Time Atomistic Observation of Structural Phase Transformations in Individual Hafnia Nanorods. *Nature Communications* **2017**, *8* (1), 15316. DOI: 10.1038/ncomms15316.
